# Phosphorylated Toll-like receptor 3 nuclear translocation in cancer cell promotes metastasis and chemoresistance

**DOI:** 10.1038/s41392-025-02307-7

**Published:** 2025-07-18

**Authors:** Zixin Wang, Yan Gu, Yanfang Liu, Ziqiao Wang, Xinyuan Chen, Haoze Wang, Wei Zhang, Gang Jin, Xuetao Cao

**Affiliations:** 1 Institute of Immunology, Zhejiang University School of Medicine, Hangzhou, China; 2 National Key Laboratory of Immunity and Inflammation, Institute of Immunology, Naval Medical University, Shanghai, China; 3 Department of Pathology, Changhai Hospital, Naval Medical University, Shanghai, China; 4 Department of Immunology, Center for Immunotherapy, Institute of Basic Medical Research, Peking Union Medical College, Chinese Academy of Medical Sciences, Beijing, China; 5 Department of Colorectal Surgery, Changhai Hospital, Naval Medical University, Shanghai, China; 6 Department of Hepatobiliary Pancreatic Surgery, Changhai Hospital, Naval Medical University, Shanghai, China; 7 Institute of Immunology, College of Life Sciences, Nankai University, Tianjin, China

**Keywords:** Tumour immunology, Molecular medicine

## Abstract

Aberrant expression and subcellular location of innate sensors in cancer cells, such as Toll-like receptors (TLRs), correlates with pro-tumoral inflammation and cancer progression, but the mechanism is still largely unknown. Deciphering the proinflammatory mediators in tumor microenvironment will contribute to the development of cancer therapeutics. By using immunohistochemistry in pancreatic ductal adenocarcinoma (PDAC) and multiple other cancer samples, here we found that cancer cell TLR3, a well-known cytoplasmic dsRNA sensor, translocated to the nucleus especially upon chemotherapy stress. Nuclear TLR3 increased the invasive and proliferative properties, and inhibited chemotherapy-induced apoptosis of cancer cells in vitro. Meanwhile, mice bearing cancer cells with nuclear TLR3 exhibited increased liver metastasis and shortened survival. Mechanistically, phosphokinase JAK1 was responsible for TLR3 phosphorylation at S155 to induce its nuclear translocation in cooperation with a nuclear transport factor importin α5. Chemotherapeutic stress induced the aberrant aggregation of dsRNA in the nucleus, which potentially contributed to nuclear TLR3 activation. Then nuclear TLR3 recruited protein arginine methyltransferase 5 (PRMT5) and bound to c-Myc to promote symmetrical dimethylation and multimerization of c-Myc, resulting in the activation of c-Myc downstream target genes and pro-tumoral signaling pathways. Accordingly, high levels of cancer cell nuclear TLR3 in clinical samples predicted patients’ worse prognosis with shorter disease-free survival, overall survival and poor response to neoadjuvant chemotherapy. Therefore, the identification of nuclear TLR3 provides new insight into non-classical functions of innate immune sensors in cancer, and JAK1/TLR3/PRMT5/c-Myc axis may sever as a potential prognostic indicator and therapeutic target to overcome chemoresistance.

## Introduction

Chronic inflammation is generally regarded as the pro-tumor factor in metastasis and chemoresistance. The pattern recognition receptors (PRRs) serve as crucial sentinels of the immune system, capable of identifying both exogenous pathogen-associated molecular patterns (PAMPs) and endogenous damage-associated molecular patterns (DAMPs).^1–3^ This recognition event quickly activates the body’s defense against danger signals, stimulating innate immunity and promoting inflammatory responses.^4–6^ Indeed, PRRs are critical activators of signaling cascades that facilitate upregulation of various inflammatory cytokines and chemokines.^4^ In addition to the critical involvement in host defense against pathogens, PRRs also participate in multiple cancer-associated cellular processes, including cancer initiation and metastasis. PRRs can recognize destabilized genomes in tumors and, paradoxically, mediate anti-tumorigenic or pro-tumorigenic effects. DAMPs, including abnormal nucleic acids in cancer cells and free DNA released by apoptotic or necrotic cancer cells, can be recognized by PRRs, activating the interferon (IFN) pathway and promoting anti-tumor immune responses.^7^ In cancers, PRRs are widely expressed in cancer cells, and also in the epithelial cells, endothelial cells and fibroblasts of the tumor microenvironment (TME).^8,9^ They even act as oncogenic proteins and promote tumor progression through non-immunological functions.^8^ However, decades of research on PRRs have predominantly focused on their activities in inducing host defense inflammatory responses. The oncogenic pathways linked to their PRR function in the TME still require further study.

Toll-like receptors (TLRs), as one of the earliest identified PRRs, are well known for pathogen recognition and innate immune activation. TLRs exhibit complex and dual roles in the anti-tumor or pro-tumor immunity. For instance, TLR agonists^10^ can trigger immune cell activation, including macrophages, dendritic cells (DCs), T cells, and natural killer (NK) cells,^11^ thus enhancing cancer immunotherapy.^12–14^ In contrast, TLRs can trigger pro-tumorigenic inflammation and provide tumor survival signals that induce tumorigenesis, promote inflammatory and immunosuppressive TME formation and cancer metastasis.^15–17^ The activation of TLRs and downstream NF-κB signaling is verified to be able to promote oncogenesis.^18,19^ Among these TLRs, TLR3, a sensor for double-stranded RNAs (dsRNA), recognizes viral dsRNA to activate the TRIF-dependent signaling pathway, initiating the host antiviral immune response in normal cellular physiology. TLR3 exerts an inhibitory effect on tumor progression, primarily through the activation of immune responses against cancer cells. TLR3 signaling induces type I interferon production, which activates NK cells and DCs, ultimately leading to tumor cell elimination through NK cell or T cell-mediated cytotoxicity.^20–22^ Paradoxically, emerging evidence suggests TLR3 may also exhibit tumor-promoting properties by enhancing malignant cell proliferation and survival signals. We once reported that the lung epithelial cell TLR3 can sense circulating tumor exosomal RNA, leading to lung chemokine production, inflammatory neutrophil recruitment and pro-metastatic niche formation to promote cancer lung metastasis.^23^ Aberrant TLR3 activation may induce proinflammatory cytokines and tumor immunosuppression. However, the pro-tumoral roles of cancer cell aberrant TLRs, particularly TLR3 expression in cancer patients and the underlying mechanisms by which TLRs act as oncogenic proteins in the TME urgently require further elucidation.

It is generally accepted that recognition of PAMPs or DAMPs by TLRs primarily occurs in the cytoplasm or endosomes. However, nucleus is known as the main site of cancer-related genetic abnormalities, including DNA damage, aberrant RNA transcription, modifications and splicing changes. We have identified a nucleus-localized DNA sensor hnRNPA2B1 which can directly recognize pathogen DNA in the nucleus to induce interferon production and elicit the immune response to clear the infection of DNA virus,^24^ suggesting that nuclear innate sensors are important on recognizing nucleic acids and triggering a downstream inflammatory responses. Indeed, a number of membrane-bound or cytoplasmic PRRs can sense nuclear changes leading to an inflammatory response once translocated to the nucleus. For example, cGAS recognizes pathogenic or DAMP DNA and translocates to the nucleus for inhibiting DNA repair through homologous recombination and promoting tumorigenesis.^25,26^ Interestingly, the expression of cytoplasmic TLR3 is reported to be a predictor of poor prognosis in breast,^27^ lung,^23^ and esophageal cancer patients^28^ and is linked to phenotypes including tumor proliferation, invasion, metastasis, and chemoresistance, but these studies have not described the subcellular location of TLR3, cytoplasm or nucleus. Our preliminary work showed that TLR3 is also located in the nucleus of cancerous cells, especially after neoadjuvant chemotherapy. However, why TLR3 locates within the nuclei of malignant cells and how chemotherapeutic agents promote nuclear translocation of TLR3 still remain poorly defined.

Here, we report that TLR3 in cancer cells undergoes nuclear translocation under chemotherapeutic stress and acts as an oncogenic protein to recruit arginine methyltransferase PRMT5 to promote dimethylation and multimerization of c-Myc, consequently facilitating cancer metastasis and chemoresistance. Phosphokinase JAK1-mediated phosphorylation of TLR3 at S155 and nuclear transport factor importin α5 are required for TLR3 nuclear translocation. Human cancer tissue analyses further confirm the correlation of high nuclear TLR3 with poor response to neoadjuvant chemotherapy and worse prognosis of patients. These findings provide new insight to the pro-tumoral effects of aberrant PRRs in cancer cells, serving as potential therapeutic targets for cancer precision therapy.

## Results

### Nuclear translocation of TLR3 in cancer cells becomes more pronounced under chemotherapeutic stress

**Fig. 1:**
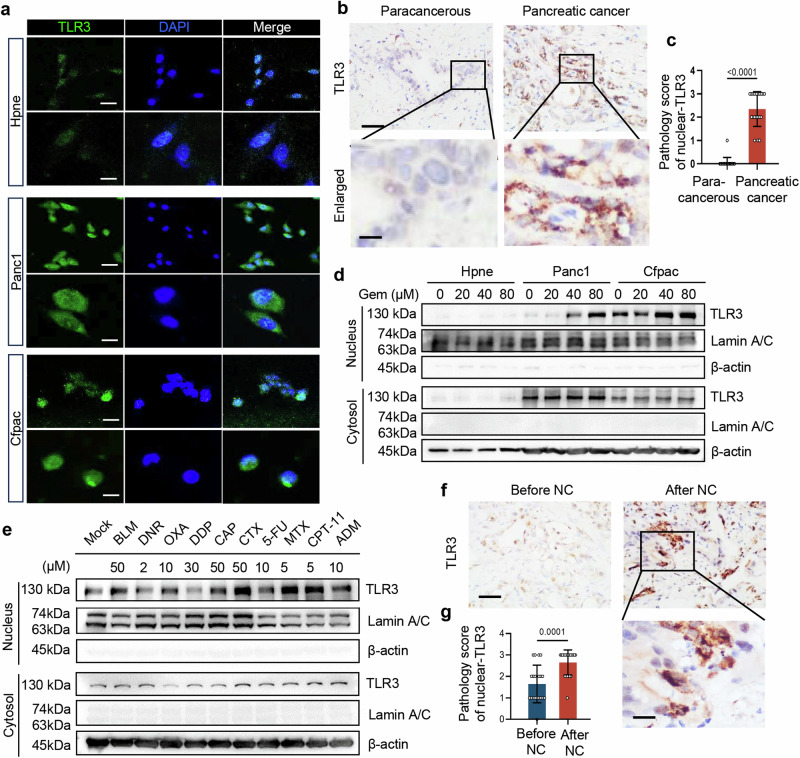
TLR3 undergoes nuclear localization in cancer especially upon neoadjuvant chemotherapy. **a** Immunofluorescent analysis of TLR3 in Hpne human pancreatic ductal cells, Panc1 and Cfpac human pancreatic adenocarcinoma cells. Scale bar (top panel), 20 μm. Scale bar (bottom panel), 10 μm. **b**, **c** Immunohistochemistry analysis (**b**) and pathology score (**c**) of TLR3 in tumor and paracancerous tissues of patients of pancreatic after neoadjuvant chemotherapy. Data shown are mean ± SD (*n* = 20). Scale bar (top panel), 50 μm. Scale bar (bottom panel), 20μm. **d** TLR3, LaminA/C and β-actin protein levels in nuclear and cytoplasmic fractions of Panc1, Cfpac cancer cells and Hpne normal cells with Gemcitabine (GEM) treatment at different concentrations (0, 20, 40, 80 μM) for 24 h. **e** TLR3, Lamin A/C and β-actin protein levels in the nucleus and cytoplasm of Panc1 cells treated with different chemotherapy drugs at the recommended cellular drug dose for cell line treatment. BLM, Bleomycin hydrochloride. DNR, Daunorubicin. OXA, Oxaliplatin. DDP, Cisplatin. CAP, Capecitabine. CTX, Cyclophosphamide. 5-FU, 5-Fluorouracil. MTX, Methotrexate. CPT-11, Irinotecan. ADM, Doxorubicin hydrochloride. **f**, **g** Immunohistochemistry analysis (**f**) and pathology score (**g**) of TLR3 in tumor tissues of pancreatic cancer patients before or after neoadjuvant chemotherapy (NC). Scale bar (top panel), 50 μm. Scale bar (bottom panel), 20μm. Data shown include the mean ± SD (*n* = 20). One representative experiment from three independent experiments is shown

To identify the role of cancer cell TLR3, we compared its expression in Panc1 and Cfpac cells (human pancreatic adenocarcinoma cell lines) with Hpne cells (human pancreatic ductal cell line) by immunofluorescence. Unexpectedly, we found nuclear localization of TLR3 in cancer cells, but barely in the corresponding normal cells (Fig. 1a and Supplementary Fig. 1a–d). Immunohistochemistry analysis confirmed TLR3 nuclear localization in pancreatic cancer, as well as colon cancer, gastric cancer and lung adenocarcinoma samples (Fig. 1b, c, and Supplementary Fig. 1e–g). To further confirm above results, we separated the cytoplasmic and nuclear protein fractions of cancer cells, and found that TLR3 was highly expressed in the cytoplasm and nucleus, especially in the nucleus of pancreatic cancer cells, as compared to that in corresponding normal cells (Fig. 1d).

We next tested potential factors driving the nuclear translocation of TLR3 in cancer cells. We found that Gemcitabine (GEM), a first-line chemotherapeutic drug broadly applied in pancreatic cancer treatment, could lead to a dose-dependent increase in TLR3 expression, as well as increased nuclear levels of TLR3 in Panc1 and Cfpac pancreatic cancer cells (Fig. 1d). We verified these results in human bronchial epithelial cell line BEAS-2B and human lung carcinoma epithelial cell A549 **(**Supplementary Fig. 1h–j**)**. These data suggest that TLR3 undergoes nuclear translocation in cancer cells but not normal cells, especially under chemotherapeutic stress.

To further examine the effects of chemotherapy on induction of TLR3 nuclear translocation, we tested other common chemotherapeutic drugs on Panc1 cells in vitro. BLM (Bleomycin), OXA (Oxaliplatin), CAP (Capecitabine), CTX (Cyclophosphamide), 5-FU (5-Flurouracil), MTX (Methotrexate) and CTP-11 (Irinotecan) all promoted nuclear translocation of TLR3 **(**Fig. 1e and Supplementary Fig. 1k). In addition, immunohistochemistry analysis of cancer tissue clinical samples confirmed that TLR3 was highly expressed in the cancer cell nucleus of tumors after neoadjuvant chemotherapy (Fig. 1f, g). These results indicate that TLR3 translocates to the nucleus in cancer cells, especially under chemotherapeutic stress.

### JAK1 induces TLR3 S155 phosphorylation to drive its nuclear translocation in cancer cells

**Fig. 2:**
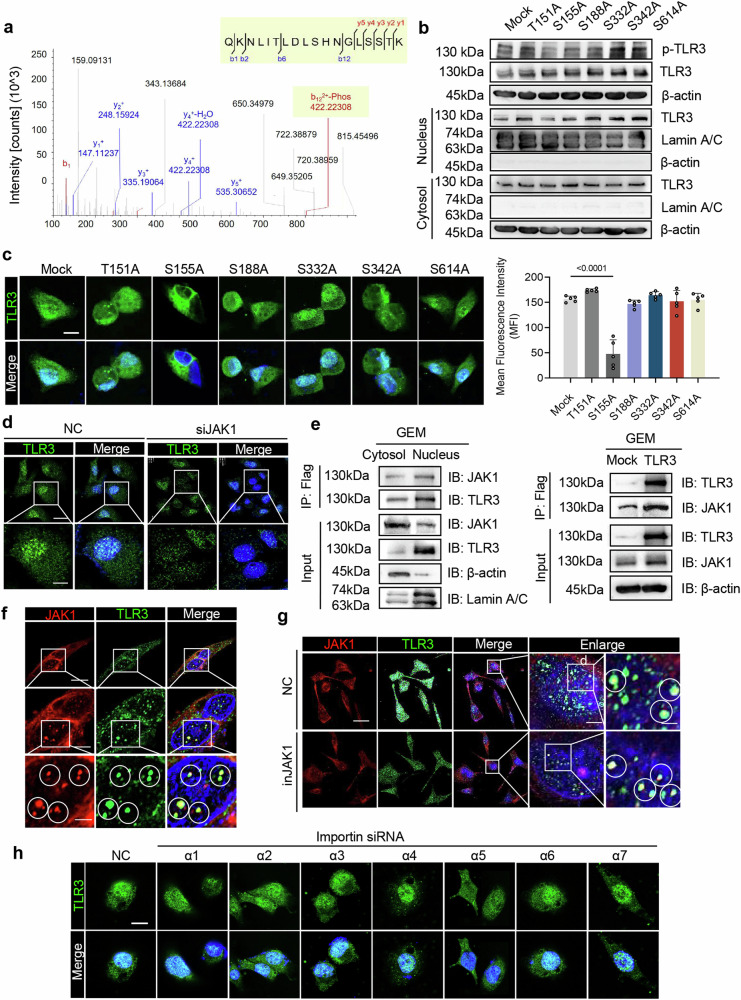
JAK1 induces TLR3 phosphorylation to drive its nuclear translocation. **a** Mass spectrometry analysis of PTMs highlights the TLR3 T151 and S155 sites. **b** Total TLR3 and TLR3 phosphorylation level, as well as TLR3 protein levels in the nucleus and cytosol of TLR3 knockout Panc1 cells transfected with TLR3 constructs carrying point mutations at T151, S155, S188, S332, S342 or S614 respectively with treatment of GEM (50 μM) for 24 h. **c** Immunofluorescent analysis and mean fluorescence indensity (MFI) of TLR3 in TLR3 KO Panc1 rescued with or without point mutation plasmids, including mutations of T151, S155, S188, S332, S342 or S614 respectively with treatment of GEM (50 μM) for 24 h. Scale bar, 5 μm. **d** Immunofluorescent analysis of TLR3 in control and JAK1-silenced Panc1 cells treated with GEM (50 μM) for 24 h. Scale bar (top panel), 20 μm. Scale bar (bottom panel), 2.5 μm. **e** Immunoblot analysis of TLR3 and JAK1 in the cytosol and nucleus of TLR3-overexpressing Panc1 cells treated with GEM (50 μM) for 24 h (left). Immunoblot analysis of TLR3 and JAK1 in WT and TLR3-overexpressing Panc1 cells treated with GEM (50 μM) for 24 h (right). **f** Immunofluorescent analysis and co-localization of JAK1 and TLR3 in Panc1 cells. Scale bar (top panel), 10 μm. Scale bar (medium panel), 2.5 μm. Scale bar (bottom panel), 1 μm. **g** Immunofluorescent analysis and co-localization of JAK1 and TLR3 in Panc1 cells with or without JAK1 inhibitor treatment. Scale bar (left panel), 50 μm. Scale bar (medium panel), 5 μm. Scale bar (right panel), 0.5 μm. **h** Immunofluorescent analysis of TLR3 in Panc1 cells with the silence of NC (negative control), Importin α1, α2, α3, α4, α5, α6, and α7 respectively. Scale bar, 10 μm

Protein phosphorylation often regulates nuclear translocation.^29,30^ To investigate the mechanisms of TLR3 nuclear translocation in cancer cells, we compared the protein phosphorylation of cytoplasmic and nuclear TLR3 using mass spectrometry (MS) after GEM treatment of Panc1 cells (Supplementary Fig. 2a). Several threonine and serine sites on TLR3 were found to be phosphorylated, including T151, S155, S188, S332, S342 and S614 **(**Fig. 2a and Supplementary Fig. 2a). Single point mutations were then constructed, including T151A (T to A at aa151), S155A (S to A at aa155), S188A (S to A at aa188), S332A (S to A at aa332), S342A (S to A at aa342) and S614A (S to A at aa614). Interestingly, rescue of TLR3 knockout Panc1 cells by TLR3-S155A resulted in a decrease in both phosphorylation level of TLR3 and nuclear TLR3 levels with or without GEM treatment (Fig. 2b, c and Supplementary Fig. 2b). These data indicate that S155 phosphorylation in the cytosol is essential for TLR3 nuclear translocation under chemotherapeutic stress.

Phosphorylation is a reversible and dynamic process that involves the balanced activities of protein kinases and phosphatases.^29^ To uncover potential phosphatases that mediate phosphorylation of TLR3, we analyzed the TLR3-associated proteins in the nucleus and cytoplasm of cancer cells by LC-MS mass spectrometry and found nine phosphorylation-related kinases among the candidates (Supplementary Fig. 2c). These nine kinases were silenced respectively in Panc1 and Cfpac pancreatic cancer cells, and then nuclear translocation of TLR3 in cancer cells was evaluated (Supplementary Fig. 2d, e). Apparently, the silence of JAK1, PAK4, RIOK1 and PKM impeded the nuclear translocation of TLR3, with more pronounced effect upon silence of JAK1 (Fig. 2d and Supplementary Fig. 3a–c).

JAK1, a key component of the interleukin-6 (IL-6)/JAK1/STAT3, plays a crucial role in affecting the expression of genes that mediate inflammation, epithelial remodeling, and metastatic cancer progression.^31–34^ Chemotherapy may activate JAK1 via JAK1-S571 phosphorylation.^35^ The interaction of TLR3 and JAK1 in Panc1 and Cfpac cells was then verified through immunoprecipitation and immunofluorescence (Fig. 2e, f and Supplementary Fig. 3d). In addition, JAK1 inhibitor resulted in a significant reduction of TLR3 in the nucleus without interfering their colocalization (Fig. 2g and Supplementary Fig. 3e). Thus, JAK1 is required for TLR3 phosphorylation and consequent nuclear translocation.

Then, we wonder whether there are cofactors for nuclear translocation of TLR3. It has been reported that importin α, comprising seven human isoforms (α1-α7), mediates substrate protein translocation across the nuclear envelope through direct binding interactions.^36^ Thus, to identify whether the importin acts as a specific cofactor for the nuclear translocation of TLR3, we depleted corresponding importin α family proteins through siRNA interference and found decreased TLR3 expression in the nucleus when importin α5 was silenced (Fig. 2h and Supplementary Fig. 3f, g). Besides, Co-Immunoprecipitation (Co-IP) assay validated the interaction of TLR3 and importin α5 (Supplementary Fig. 3h). Thus, importin α5 is the cofactor for TLR3 nuclear translocation.

Cancer cell genomes exhibit unstable characteristics including aberrant RNA expression such as double-stranded dsRNA, especially under chemotherapeutic treatment. Then we identified the role of nuclear dsRNA accumulation on TLR3 translocation and activation, Co-localization of TLR3 and dsRNA detected by J2 antibody was observed in the nucleus of Panc1, Cfpac and A549 cells under chemotherapeutic stimulation. Notably, dsRNA accumulated within the nuclei of cancer cells in a dosage dependent, subsequent to treatment with GEM (Supplementary Fig. 4a–c). Time-course analysis indicated that dsRNA accumulated in the nucleus and co-localization with TLR3 starting at 12 hours post-administration of GEM (Supplementary Fig. 4d). Furthermore, we pre-treated cells with CU-CPT 4a to competitively inhibit dsRNA/TLR3 binding to determine the functional impact of dsRNA on TLR3 nuclear dynamics, and found that CU-CPT 4a did not affect the nuclear translocation of TLR3 (Supplementary Fig. 4e). These data demonstrate that dsRNA accumulation in the nucleus triggers TLR3 activation after TLR3 nuclear translocation.

### Nuclear TLR3 promotes cancer cell invasion and chemoresistance

**Fig. 3:**
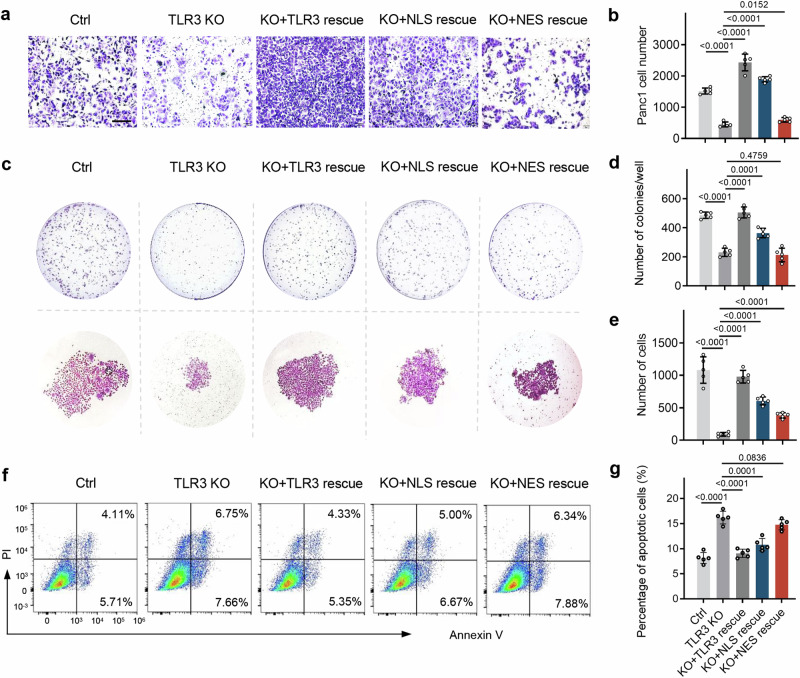
Nuclear TLR3 promotes cancer cell proliferation, invasion and inhibits apoptosis. **a**, **b** Representative images of colonies and quantification of cell numbers in WT and TLR3 KO Panc1 cells rescued with TLR3, NLS-TLR3, or NES-TLR3 in a transwell invasion assay. Scale bar, 50 μm. Data shown are mean ± SD (*n* = 5). **c**–**e** Colony formation was determined in WT, TLR3 KO, TLR3 rescue, NLS-TLR3 rescue, NES-TLR3 rescue Panc1 cells (**c**). Colony numbers are shown in the bar graph (**d**, **e**). Data shown are mean ± SD (*n* = 5). (**f**, **g**) The proportions (**f**) of WT Panc1 cells, or Panc1 cells with TLR3 KO, TLR3 rescue, NLS-TLR3 rescue, NES-TLR3 rescue after treatment with GEM (200 μM, 24 h) were detected by flow cytometry. The percentage of cells in apoptosis is shown in the bar graph (**g**). Data shown are mean ± SD (*n* = 5). One representative experiment from three independent experiments is shown

To investigate the role of nuclear TLR3 of cancer cells, we rescued TLR3 knockout Panc1 cells by expressing wild-type TLR3, the TLR3 with a nuclear localization signal (NLS-TLR3), or the TLR3 with a nuclear export signal (NES-TLR3) (Supplementary Fig. 5a–f). An invasion assay indicated that TLR3 knockout significantly reduced the invasive properties of the cancer cells, while rescue with NLS-TLR3 led to a more invasive phenotype compared to NES-TLR3 (Fig. 3a, b, and Supplementary Fig. 6a, b). The clone formation assay showed that the rescue with NLS-TLR3 greatly restored cancer cell proliferation with formation of more cell colonies compared with NES-TLR3 (Fig. 3c–e and Supplementary Fig. 6c–e).

Previous studies have shown that cytoplasmic dsRNA triggers cytosolic sensing by TLR3 and activates downstream pathways, leading to the type I interferon response and tumor cell apoptosis.^37–39^ For the effect of nuclear TLR3 on the cancer cell apoptosis, we detected the apoptotic ratio of cancer cells with TLR3 knockout or TLR3 rescue upon GEM treatment. We found an obvious reduction in the number of apoptotic TLR3 knockout cancer cells, compared to control cancer cells. Moreover, rescue with NLS-TLR3 led to a reduction of cancer cell apoptosis compared to rescue of TLR3 with NES-TLR3 (Fig. 3f, g and Supplementary Fig. 6f, g).

Collectively, nucleus-localized TLR3 promotes cancer cell proliferation, invasion and contributes to chemoresistance by inhibiting cancer cell apoptosis.

### Nuclear TLR3 promotes tumor growth and metastasis in vivo

**Fig. 4:**
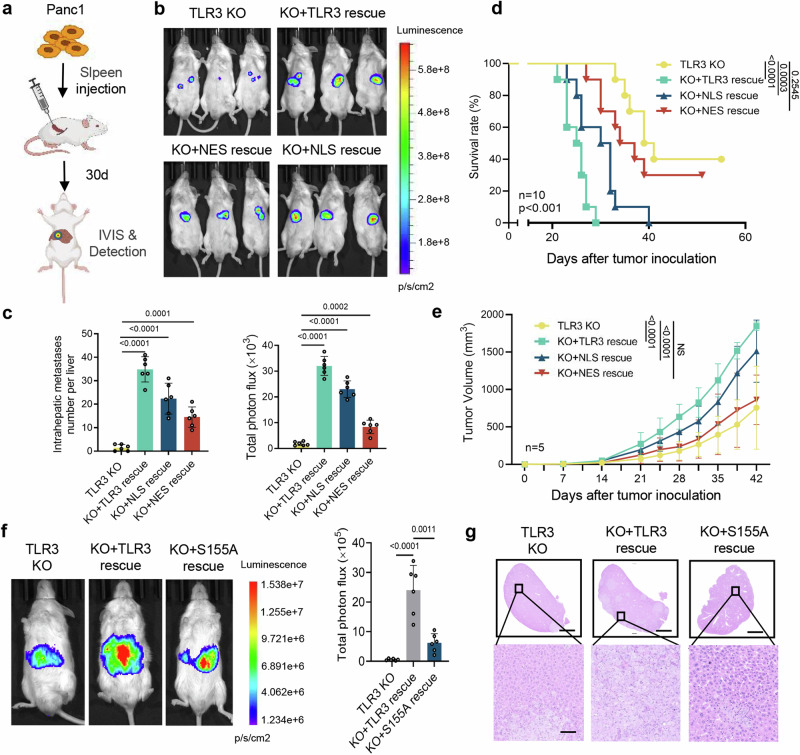
Nuclear TLR3 promotes tumor metastasis and decreases survival of tumor-bearing mice. **a** Schematic showing analysis of Panc1 cell metastasis in a mouse model after splenic injection. **b** Representative images of liver metastasis in NSG mice with splenic injection of Panc1 cells with TLR3 knockout, wild-type TLR3, NLS-TLR3, or NES-TLR3 rescue respectively as detected by luciferase-based bioluminescence imaging. **c** Quantification of liver metastasis foci in NSG mice with splenic injection of Panc1 cells with TLR3 knockout, wild-type TLR3, NLS-TLR3, or NES-TLR3 rescue are shown in the bar graph. Data shown are mean ± SD (*n* = 6). **d** Survival of NSG mice with splenic injection of Panc1 cells with TLR3 knockout, wild-type TLR3, NLS-TLR3, or NES-TLR3 rescue. Kaplan–Meier test (*n* = 10). **e** Tumor volume over the course of the study in NSG mice with subcutaneous injection of Panc1 cells with TLR3 knockout, wild-type TLR3, NLS-TLR3, or NES-TLR3 rescue (*n* = 5). *P* values, two-way analysis of variance (ANOVA). **f**, **g** Representative images and quantitative analysis of liver metastasis in NSG mice with splenic injection of Panc1 cells with TLR3 knockout, wild-type TLR3 or S155A-TLR3 rescue respectively as detected by luciferase-based bioluminescence imaging (**f**) and H&E-stained liver sections (**g**). Data shown are mean ± SD (*n* = 6). One representative experiments from three independent experiment is shown

To clarify the in vivo functions of nuclear TLR3, we constructed a tumor-bearing mouse model with pancreatic cancer liver metastasis. Panc1 cells with TLR3 knockout, wild-type TLR3 rescue, NLS-TLR3 rescue or NES-TLR3 rescue were inoculated into the spleen of immunodeficient NSG (NOD.Cg-Prkd^cscid^ IL2rgtm^1W*jl*^/SzJ) mice (Fig. 4a). We found that TLR3 knockout led to a remarkable decrease in liver metastasis in tumor-bearing mice, which was rescued by wild-type TLR3 or NLS-TLR3 rescue, but not NES-TLR3 rescue (Fig. 4b, c and Supplementary Fig. 7a–c). Accordingly, the tumor-bearing mice inoculated with TLR3 knockout cancer cells rescued with NES-TLR3 survived much longer than that with wild-type TLR3 or NLS-TLR3 rescue (Fig. 4d). To further validate the pro-tumoral phenotypes of nuclear TLR3 in immunocompetent mice, TLR3 knockout mouse pancreatic cancer cell lines (PanO2) were constructed and pancreatic cancer liver metastasis models in immunocompetent C57BL/6 mice were established. Compared to NES-TLR3 rescue, NLS-TLR3 rescue induced more significant metastasis, which was consistent with the precious conclusion in NSG mice (Supplementary Fig. 7d–f). These results indicate that the nucleus-localized TLR3 promotes cancer metastasis.

To test whether TLR3 affects tumor growth in vivo, Panc1 cells were inoculated into the NSG mice subcutaneously. We found that the growth of cancer cells with TLR3 knockout was significantly decreased, which was rescued by wild-type TLR3 or NLS-TLR3 rescue, but not NES-TLR3 rescue, demonstrating the contribution of nucleus-localized TLR3 in promoting tumor progression (Fig. 4e and Supplementary Fig. 7g). For the role of S155 phosphorylation in TLR3, we inoculated TLR3 knockout Panc1 cells with wild-type TLR3 or S155A TLR3 rescue into the spleen of immunodeficient NSG mice. Apparently, rescue of TLR3 knockout cells by expressing S155A TLR3 resulted in a decrease in liver metastasis (Fig. 4f, g and Supplementary Fig. 7h), indicating the importance of S155 phosphorylation of TLR3 on facilitating metastasis.

Taken together, these results suggest that nuclear TLR3 in cancer cells promotes tumor growth and metastasis.

### Nuclear TLR3 interacts with c-Myc and promotes c-Myc multimerization in cancer cells

**Fig. 5:**
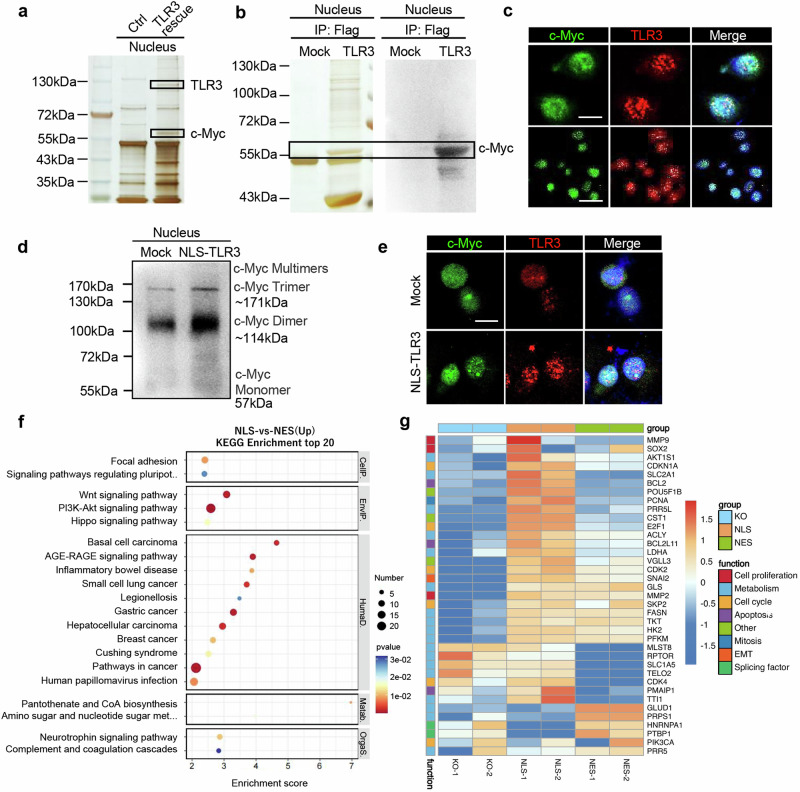
Nuclear TLR3 promotes c-Myc multimerization in cancer cells. **a** Proteins pulled down from TLR3-knockout Panc1 cells with or without TLR3 rescue were visualized by silver staining. **b** Silver staining (left panel) and immunoblots (right panel) showing interaction between c-Myc and nuclear TLR3 in WT and TLR3-overexpressing Panc1 cells. **c** Immunofluorescent analysis and co-localization of c-Myc and TLR3 in Panc1 cells. Scale bar (top panel), 10 μm. Scale bar (bottom panel), 40 μm. **d** Immunoblot analysis of c-Myc multimers in the nucleus of WT and NLS-TLR3 overexpressed Panc1 cells after GEM (50 μM) stimulation for 24 h. **e** Immunofluorescent analysis of c-Myc multimers and TLR3 in NLS-TLR3 overexpressed Panc1 cells with GEM (50 μM) stimulation for 24 h. Scale bar, 10 μm. **f** Top 20 pathways that were significantly upregulated in NLS-TLR3 rescue Panc1 cells compared with NES-TLR3 rescue via KEGG enrichment. cutoff, *p* < 0.05. **g** The heatmap of hallmarker gene sets of Panc1 cells with TLR3 knockout, NLS-TLR3 or NES-TLR3 rescue. One representative experiment from three independent experiments is shown

To uncover the mechanisms underlying the pro-tumoral function of nuclear TLR3, we performed LC-MS mass spectrometry to identify TLR3-binding proteins in the nucleus of cancer cells. Unexpectedly, our data indicated oncogene c-Myc as a TLR3-binding protein (Fig. 5a). c-Myc is activated in the great majority of cancers.^40–42^ As a transcription factor,^43,44^ c-Myc regulates various biological programs including tumor immune escape and pathophysiological dysregulation in the TME.^45^ We validated TLR3 interaction with c-Myc in Panc1 cells (Fig. 5b, c) and in A549 cells (Supplementary Fig. 8a). But both the transcriptional and protein levels of c-Myc showed no significant difference in established cell lines including Panc1 cells with TLR3 knockout, wild-type TLR3, NLS-TLR3 or NES-TLR3 rescue (Supplementary Fig. 8b, c).

It has been reported that c-Myc can form spherical multimeric structures with binding sites in active promoters and block antisense transcription, thus contributing to tumor cell proliferation under cell stress.^46^ Therefore, we speculated that the accumulation of nuclear TLR3 might alter c-Myc multimerization. As previously reported, c-Myc appeared to form multimeric structures in the nucleus, as verified by Native PAGE and immunofluorescence (Fig. 5d, e). Interestingly, NLS-TLR3 overexpression could promote the multimerization of c-Myc in cancer cells (Fig. 5d, e).

To further show the impact of NLS-TLR3 on the functions of c-Myc, RNA-seq of Panc1 cells with TLR3 knockout, NES-TLR3 or NLS-TLR3 rescue was performed. Key downstream signaling pathways of c-Myc, such as Wnt, PI3K-Akt and Hippo signaling pathways were found to be upregulated in Panc1 cells with NLS-TLR3 rescue (Fig. 5f), as confirmed by WikiPathways (Supplementary Fig. 8d). Accordingly, target genes of c-Myc (such as *Cst1*, *Snai2* and *Bcl2*) related to tumor invasion, metastasis, and drug resistance were upregulated in Panc1 cells with NLS-TLR3 rescue, compared with other two groups (Fig. 5g). CUT&Tag qPCR further confirmed that c-Myc could bind to the promoter of Cst1, Snai2 and Bcl2, which was decreased in TLR3 KO Panc1 cells (Supplementary Fig. 8e). Thus, nuclear TLR3 promotes the multimerization of c-Myc and subsequent activation of its downstream target genes and related signaling pathways.

### Nuclear TLR3 recruits PRMT5 to mediate c-Myc dimethylation and multimerization in cancer cells

To analyze how the nuclear TLR3 impacts c-Myc multimerization, we checked the potential TLR3-binding proteins identified by MS and noticed protein arginine methyltransferase 5 (PRMT5). PRMT5 methylates various substrates including p53 and histones,^47^ and participates in DNA repair, cell cycle regulation, and transcriptional control.^48–50^ PRMT5, as an epigenetic enforcer, upregulated c-Myc expression in gastric cancer, synergistically promotes cancer cell proliferation and inhibits apoptosis.^51^

**Fig. 6:**
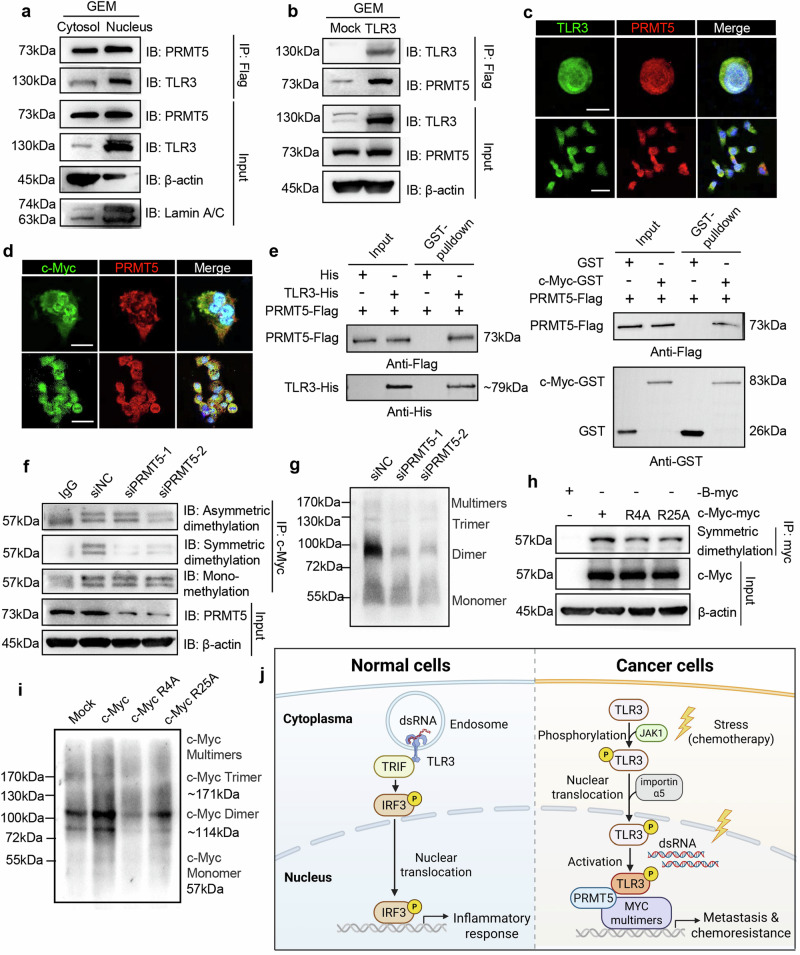
Nuclear TLR3 recruits PRMT5 to induce the symmetric dimethylation and multimerization of c-Myc. **a** Immunoblot analysis of TLR3 and PRMT5 in the cytosol and nucleus of TLR3 overexpressing Panc1 cells treated with GEM (50 μM) for 24 h. **b** Immunoblot analysis of TLR3 and PRMT5 in WT and TLR3 overexpressing Panc1 cells treated with GEM (50 μM) for 24 h. **c** Immunofluorescent analysis of TLR3 and PRMT5 in Panc1 cells treated with GEM (50 μM) for 24 h. Scale bar (top panel), 10 μm. Scale bar (bottom panel), 40 μm. **d** Immunofluorescent analysis of c-Myc and PRMT5 in Panc1 cells. Scale bar (top panel), 10 μm. Scale bar (bottom panel), 30 μm. **e** Immunoblot analysis of PRMT5-Flag binding to purified His control and TLR3-His (24-702aa) using Flag-tag antibody (top) and His-tag antibody (left). Immunoblot analysis of PRMT5-Flag binding to purified GST and c-Myc-GST using Flag-tag antibody (top) and GST-tag antibody (right). **f** Immunoblot analysis of asymmetric dimethylation, symmetric dimethylation, and mono-methylation of c-Myc in siNC and PRMT5-silenced Panc1 cells. **g** Immunoblot analysis of c-Myc multimers in siNC and PRMT5-silenced Panc1 cells. **h** Immunoblot analysis of symmetric dimethylation of c-Myc in Panc1 cells with wild-type c-Myc, R4A and R25A c-Myc overexpression. **i** Immunoblot analysis of c-Myc multimers in Panc1 cells with wild-type, R4A or R25A c-Myc overexpression. One representative experiment from three independent experiments is shown. **j** Schematic diagram, edited by BioRender.com, indicates that cytoplasmic TLR3 in normal cells induces homeostatic inflammatory response but the nuclear translocated TLR3 in cancer cells interacts with c-Myc to promote tumor metastasis and chemoresistance under chemotherapeutic stress

We verified the interaction between TLR3 and PRMT5 in the nucleus of Panc1 and A549 cancer cells upon chemotherapeutic stress by immunoprecipitation and immunofluorescence (Fig. 6a–c and Supplementary Fig. 8f). The interaction between c-Myc and PRMT5 in the nucleus was also confirmed, which can be increased in Panc1 cells with GEM treatment, but decreased in TLR3 knockout Panc1 cells (Fig. 6d and Supplementary Fig. 8g–j). GST-pulldown assay showed that TLR3 could directly bind to PRMT5, and also PRMT5 could directly bind to c-Myc (Fig. 6e). Moreover, the symmetric dimethylation of c-Myc was reduced in PRMT5-silenced cancer cells (Fig. 6f).

As expected, c-Myc multimerization was reduced by silencing of PRMT5 (Fig. 6g and Supplementary Fig. 9a**)**. Then, MS was performed to identify c-Myc methylation sites, indicating R4 and R25 as potential sites of dimethylation in c-Myc (Supplementary Fig. 9b, c). Single point mutations were then constructed, including R4A (R to A at aa4) and R25A (R to A at aa25), and the dimethylation in c-Myc was decreased in Panc1 cells overexpressed with R4A or R25A mutations (Fig. 6h). Interestingly, R4A mutation of c-Myc obviously led to the decline in its multimerization (Fig. 6i), indicating that nuclear TLR3 recruits PRMT5 to mediate c-Myc dimethylation, contributing to the increased expression and multimerization of c-Myc in the nucleus.

To reinforce the roles of c-Myc/PRMT5 as downstream effectors, we used c-Myc inhibitor or PRMT5 inhibitor for in vivo treatment in the pancreatic cancer liver metastasis model bearing TLR3 knockout cancer cells with NLS-TLR3 rescue. Consistent with the previous work, TLR3 rescue contributed to a remarkable increase in liver metastasis in tumor-bearing mice, while either c-Myc inhibitor or PRMT5 inhibitor suppressed the metastasis induced by TLR3 rescue (Supplementary Fig. 9d, e).

### Elevated nuclear TLR3 expression in cancer cells correlates with tumor progression, chemoresistance, and patients’ poor outcomes

**Fig. 7:**
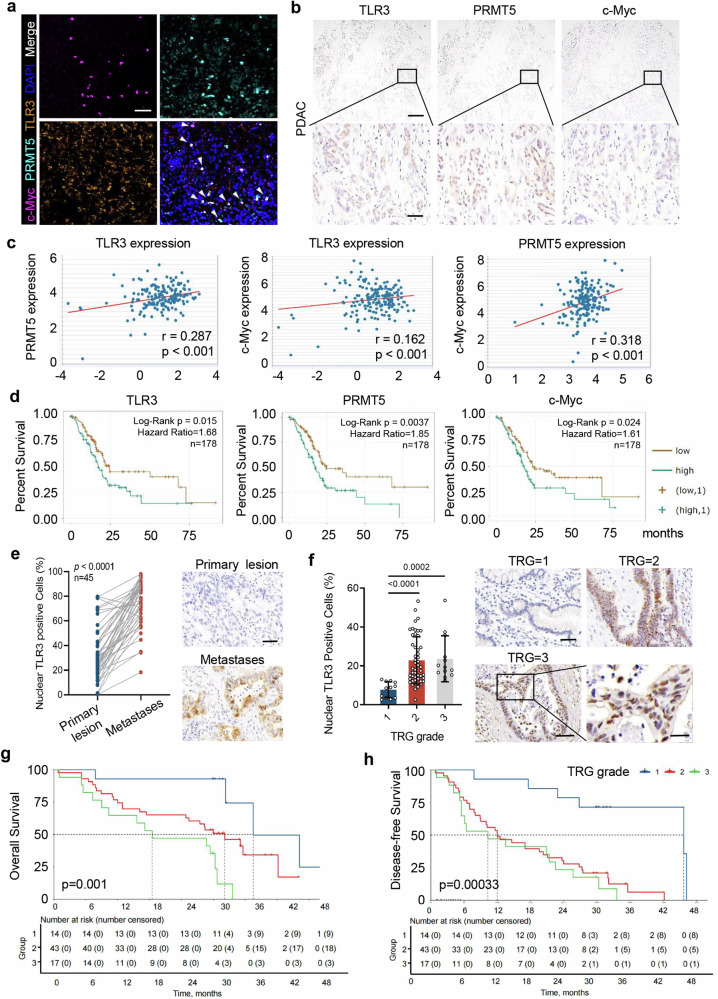
Nuclear TLR3 is correlated with PRMT5 and c-Myc expression and its high level predicts patients’ poor prognosis and chemoresistance. **a** Immunofluorescent analysis of c-Myc, TLR3 and PRMT5 expression in tumor tissue samples from pancreatic cancer patients after neoadjuvant chemotherapy. Scale bar, 150 μm. **b** Immunohistochemistry analysis of TLR3, PRMT5 and c-Myc expression in tumor tissues of pancreatic cancer patients after neoadjuvant chemotherapy. Scale bar (top panel), 150 μm. Scale bar (bottom panel), 20 μm. **c** Scatterplot of two-two corelations between TLR3 and PRMT5, TLR3 and c-Myc, PRMT5 and c-Myc at the transcriptional level (expression of log2(FPKM + 0.01)) in pancreatic cancer (*n* = 178) (ENCORI, starBase V3.0). **d** Overall survival of pancreatic cancer patients with low or high expression of TLR3, PRMT5 and c-Myc, respectively (*n* = 178) (ENCORI, starBase V3.0). Kaplan–Meier test. **e** Immunohistochemical analysis and representative images of nuclear TLR3-positive cancer cells in primary lesions and the corresponding metastasis from pancreatic cancer patients (*n* = 45). Scale bar, 50 μm. *P* values, paired samples *t* test. **f** Immunohistochemical analysis and representative images of nuclear TLR3 positive cancer cells in tumor tissue samples from pancreatic cancer patients after neoadjuvant chemotherapy. Scale bar, 50 μm (top panel). Scale bar, 15 μm (bottom panel). TRG, pathological tumor regression grade. TRG = 1, single cells or small groups of tumor cells; TRG = 2, residual tumor cells with desmoplastic response; TRG = 3, minimal evidence of tumor response. *P* values, student’s two-tailed *t* test. **g**, **h** Overall survival and disease-free survival of pancreatic cancer patients with different tumor regression grades after neoadjuvant chemotherapy. Kaplan–Meier test

To clarify the clinical relevance between TLR3, PRMT5 and c-Myc in clinical samples, immunofluorescent analysis of TLR3, PRMT5 and c-Myc was performed. Co-localization of these three molecules within the nuclei of cancer cells presented in tumor tissue sections obtained from pancreatic cancer patients undergoing neoadjuvant chemotherapy (Fig. 7a). Furthermore, we employed pancreatic cancer tissue microarrays to analyze the expression patterns of TLR3, PRMT5 and c-Myc (Fig. 7b). Moreover, analysis of RNA co-expression using ENCORI (starBase V3.0) databases indicated a positive correlation among these molecules at the transcriptional level (Fig. 7c). Additionally, high levels of TLR3, PRMT5 and c-Myc transcripts were correlated with reduced survival rates in pancreatic cancer patients (Fig. 7d).

Furthermore, we performed immunohistochemical staining to detect TLR3 expression in primary and corresponding metastatic lesions from 45 pancreatic cancer patients. Notably, an obvious increase of nuclear TLR3 was observed in lymph node metastasis relative to primary lesions (Fig. 7e). Additionally, using a separate cohort of 74 pancreatic cancer patients who underwent neoadjuvant chemotherapy prior to surgical resection, we found that patients with a high tumor regression grade (TRG) showed a significantly increased nuclear TLR3 expression in cancer cells (Fig. 7f). Accordingly, patients with higher cancer cell nuclear TLR3 expression had shorter overall survival (OS) and disease-free survival (DFS) after surgery (Fig. 7g, h).

Consistent with mouse studies, these clinical data demonstrate a correlation between elevated nuclear TLR3 expression in cancer cells and the increased progression, chemoresistance, poor prognosis of cancer patients.

## Discussion

The aberrant expression and activation of TLRs in cancer cells are reported to be closely associated with inflammation-related neoplastic progression. In this study, we report that TLR3, a traditionally membrane-associated PRR in the cytoplasm, translocates to the nucleus of cancer cells, in particular under chemotherapeutic stress. Nuclear translocated TLR3 may be linked to the recognition of dsRNA aggregated in cancer cell nucleus upon chemotherapeutic stress and bridges TLR3/PRMT5/c-Myc interaction to trigger symmetric dimethylation and multimerization of c-Myc, and the molecular events contribute to cancer metastasis and chemoresistance (Fig. 6j). These findings propose a previously unknown function of nuclear TLR3 in cancer both basically and clinically, providing new insights into the mechanisms underlying chemoresistance. Our data suggest that cancer cell expression level of nuclear TLR3 may help the prediction of cancer patient prognosis and provide a prospective target for cancer treatment.

The traditional function of dsRNA sensor TLR3 is to mediate host inflammatory innate responses by inducing inflammatory cytokines and type I IFN expression via recruiting TIR structural domain-containing adapter protein (TRIF). On the other hand, TLR3 shows significant upregulation, within both neoplastic and stromal compartments across various malignancies including breast, lung or esophageal cancers, in which it regulates cancer cell proliferation, invasion and apoptosis.^52,53^ Interestingly, our investigations revealed a novel nuclear localization pattern of TLR3 in malignant cells. Chemotherapeutic intervention induced marked elevation of total TLR3 expression in cancer cells, and the increased nuclear TLR3 may result from the increased expression level of total TLR3 and its translocation to the nucleus. In this process, JAK1 plays an essential role to induce TLR3 S155 phosphorylation and drive its nuclear translocation. Recent attention has been drawn to the roles of JAK inhibitors on improving responses to immune checkpoint inhibitors in cancer immunotherapy.^54,55^ Building upon established findings that immunosuppressants like Rapamycin and Cyclosporine A can modulate tumor immunogenicity by suppressing inflammatory responses,^56–58^ we wonder whether JAK1 inhibitor could act on tumor cells and increase their responses to immunotherapy. Our investigations indicated that JAK1 inhibitor treatment effectively suppressed TLR3 nuclear translocation in malignant cells while maintained TLR3 colocalization patterns, suggesting the critical role of JAK1 on triggering TLR3 downstream pro-tumoral function. Thus, we supposed that pharmacological JAK1 inhibition could improve immunotherapeutic outcomes via blockade of TLR3 nuclear translocation and pro-tumoral function. Moreover, functioning as a critical nuclear transport adaptor, the α subunit of importin directly engages substrate proteins via its armadillo repeat domain, forming high-affinity complexes that facilitate active translocation across the nuclear envelope.^59^ However, how chemotherapy activates JAK1 accompanied with co-factor importin α5 to potentiate TLR3 phosphorylation and translocation needs further investigation. Furthermore, dsRNA accumulated within the nuclei of cancer cells in a dosage-dependent way post the treatment of GEM, and the dsRNA/TLR3 competitive binding inhibitor did not affect the nuclear translocation of TLR3. Why dsRNA accumulates in the nucleus and how TLR3 interacts with nuclear dsRNA in cancer cells requires future analysis.

The nuclear TLR3 is found to be able to recruit the arginine methyltransferase PRMT5 to promote symmetrical dimethylation and multimerization of c-Myc in cancer cells. Transgenic overexpression of c-Myc^60–62^ and activation of c-Myc tend to elicit certain hallmarks of cancers including tumorigenesis, tumor proliferation, as well as invasiveness, angiogenesis and immune evasion.^63–65^ The symmetric dimethylation and multimerization of c-Myc was recently reported to enable cancer cells to proliferate under stressful conditions, but the underlying mechanisms remained unclearly defined.^46^ Here, our investigations revealed that nuclear TLR3 activation played a functional role in stimulating c-Myc-regulated target genes and associated signaling cascades such as Wnt, PI3K-Akt and Hippo signaling pathways. PRMT5, a principal type II arginine methyltransferase, catalyzes symmetric dimethylation of arginine residues in both histone and non-histone proteins.^66–69^ Subsequent analyses demonstrated significant co-expression patterns among TLR3, PRMT5, and c-Myc in malignant cells, with concordant observations at both transcriptional and protein expression levels, especially the direct binding between TLR3 and PRMT5, as well as PRMT5 and c-Myc. These findings highlight a previously unknown function of nuclear TLR3 in the modifications and multimerization of c-Myc for promoting tumor progression. Meanwhile, c-Myc multimers protect stalled replication forks and restrict the formation of double-strand breaks thus enhancing chromosome stability,^46^ suggesting that the multimerization of c-Myc potentially serves a vital function in genome stability of tumors, which contributes to chemoresistance especially under cellular stress conditions. Besides, c-Myc inhibitor and PRMT5 inhibitor both suppressed the metastasis induced by TLR3 rescue in the pancreatic cancer liver metastasis model bearing TLR3 knockout cancer cells, reinforcing the non-redundant role of c-Myc/PRMT5 as downstream effectors. However, additional clarification is required regarding the functions and molecular mechanisms underlying the interactions between TLR3, PRMT5 and c-Myc. How dimethylation of c-Myc affects its multimerization is one open question for future in-depth investigation.

Clinically, we found the co-localization of TLR3, PRMT5 and c-Myc within the nuclei of cancer cells presented in tumor tissue sections obtained from pancreatic cancer patients undergoing neoadjuvant chemotherapy. Besides, increased levels of nucleus-localized TLR3 positively correlated with poor histological differentiation and vascular thrombosis of cancer patients, and negatively correlated with patients’ survival. Interestingly, the patients with high TRG after neoadjuvant therapy had a higher percentage of cancer cells with TLR3 nuclear expression, accompanied with worse clinical outcomes and shortened survival. Previous work mainly focused on cytoplasmic TLR3 to facilitate tumor progression,^70^ metastasis^52^ or inhibit anti-tumor immunity^71^ in various cancers. Our findings reveal a new way by which nucleus-localized TLR3 contributes to chemoresistance and poor prognosis of cancer via interactions with oncogenic PRMT5 and c-Myc in the nucleus. Although we verified our observations in clinical samples from pancreatic cancer and several other cancers, more types of cancers are required to verify the pro-tumoral functions of nuclear TLR3 in pan-cancer research. Also, strategies for identifying molecules that synergize with TLR3 as potential tumor therapeutic targets still warrants deeper exploration.

Taken together, our study uncovers a non-canonical function of nuclear TLR3 to act as an oncogenic protein that drives tumor metastasis and chemoresistance. The JAK1/TLR3/PRMT5/c-Myc axis provides potential targets for cancer and reasonable evidence for predicting chemotherapeutic efficacy and patients' prognosis.

## Materials and methods

### Clinical samples

Human pancreatic cancer tissue microarrays (HPanA170Su04-M-023) were provided by Shanghai Outdo Biotech Co. Ltd. Immunofluorescence and Immunohistochemistry staining targeting TLR3 were conducted on the tumor tissues and lymph nodes derived from patients diagnosed with PDAC. A total of 45 cases were examined to compare primary lesions and lymph node metastases. An independent cohort comprising 74 surgical specimens from patients who had undergone neoadjuvant therapy was acquired from Changhai Hospital. These clinical samples, collected between 2018 and 2020 at Changhai Hospital affiliated with Naval Medical University (Shanghai, China), originated exclusively from patients receiving preoperative neoadjuvant treatment. Immunohistochemical analyses were conducted utilizing the Pannoramic DESK/MIDI/250 FLASH digital slide scanner (3DHISTECH) facilitated by Wuhan Servicebio Technology Co., Ltd. In accordance with ethical guidelines, written informed consent was procured from each participant enrolled in this investigation. The ethics committee of Changhai Hospital granted approval for the conduct of this study (CHEC2024-109).

### Mice and cells

NSG Immunodeficient mice (NOD.Cg-Prkdcscid IL2rgtm1Wjl/SzJ) and C57BL/6 mice with age-matched (6-8 weeks) and gender-balanced were procured from GemPharmatech Co., Ltd. The mice were bred under specific pathogen-free (SPF) conditions throughout our experiment. Experimental procedures involving mice were conducted following guidelines approved by the ethics committee of Naval Medical University (NMUEC2023-0125), Shanghai.

Hpne, Panc1, Cfpac and A549 cell lines were obtained from American Type Culture Collection (ATCC). Hpne, Panc1 and Cfpac cells were cultured in DMEM/F12 medium supplemented with 10% fetal bovine serum (FBS, 10099141 C, Gibco) and A549 was cultured in DMEM medium supplemented with 10% FBS.

### Preparation of tumor-bearing mice

In liver metastatic models, 1×10^6^ luciferase-labeled Panc1 cells with TLR3 knockout, the rescue of wild-type TLR3, NLS-TLR3, or NES-TLR3 were injected into the spleen of NSG mice. Metastasis progression in the liver was confirmed over a 30-day period via ex vivo bioluminescence imaging using the IVIS Lumina II system (Perkin-Elmer), with definitive histological validation through H&E-stained paraffin sections.

In the subcutaneous tumor model, 1×10^6^ Panc1 cells with TLR3 knockout, the rescue of TLR3, NLS-TLR3, or NES-TLR3 were inoculated into the dorsum of NSG mice subcutaneously. Tumor size was assessed with a caliper twice a week from D7 until the humane endpoints were reached.

In liver metastatic models treated with the c-Myc or PRMT5 inhibitor, 1 × 10^6^ luciferase-labeled TLR3 knockout Panc1 cells and NLS-TLR3 rescue Panc1 cells were inoculated in the spleen of NSG mice. Mice were administrated with the EPZ015666 (200 mg/kg) by oral gavage daily for 3 weeks or treated i.v. with 10058-F4 (30 mg/kg) daily for 5 days per week for 3 weeks. The liver metastasis was detected approximately 30 days after the inoculation by IVIS and were subsequently confirmed through histological examination of H&E-stained paraffin-embedded tissue sections.

### Cellular segregation of nuclear and cytoplasmic components

Cellular fractionation was carried out to isolate nuclear and cytoplasmic components utilizing the NE-PER extraction system (Thermo Fisher, Cat #78833) following the recommended protocol. The related reagents and resources are listed in Table S1.

### Cell transfection

For plasmid transfection, cells were transfected at a final concentration of 1 µg/100 µl using X-treme GENE™ HP (06366236001, Sigma-Aldrich). For gene silencing experiments, cells were treated with 10 µM siRNA complexed with Lipofectamine RNAiMAX transfection reagent (13778150, Thermo Fisher). siRNAs were from Shanghai GenePharma Co., Ltd. The primers for PCR in plasmid construction are listed in Table S2. TLR3 CRISPR guide RNA sequences in plasmid construction are listed in Table S3. The sequences of siRNAs are listed in Table S4, and primers for RT-qPCR are listed in Table S5.

### Cell invasion assay

Cellular invasion assays were conducted using modified chambers (Corning Inc, USA) with 8 μm polycarbonate membranes coated by Matrigel matrix. For the cell migration study, the upper chamber of a transwell system was coated with 100 μL of Matrigel and seeded with 1 × 10^5^ cells in 200 μL of serum-free DMEM. The lower chamber received 500 μL of DMEM supplemented with 10% FBS as a chemoattractant, and the system was incubated for 24 h at 37 °C in a 5% CO_2_. Methanol-fixed cells (30 min treatment) were visualized by 0.4% crystal violet staining and imaged with an Olympus IX70 microscope. Migration was quantified in three independent experiments using ImageJ analysis.

### Immunohistochemical staining

Immunohistochemical analysis was conducted on paraffin-embedded sections fixed with paraformaldehyde. Primary antibodies against TLR3 (#6961), JAK1 (#3344), PRMT5 (#79998), and c-Myc (#18583) from Cell Signaling Technology (CST) were utilized for immunohistochemical analysis employing a streptavidin-peroxidase conjugate-based immunohistochemistry (SP-IHC) method. The immunohistochemical procedures and evaluation were carried out following previously published protocols^17^. Two pathologists conducted independent evaluations of all surgical pathology specimens. Quantitative assessment of immunostaining was performed by evaluating both the proportion of labeled cells (scored from 1% to 100%) and staining intensity (graded on a 4-point scale: 0 = absent, 1 = faint, 2 = moderate, 3 = intense). Chemotherapeutic efficacy was determined through pathological evaluation using the tumor regression grading (TRG) system, where: TRG 0 indicated complete pathological response (pCR) with no viable tumor cells; TRG 1 represented minimal residual disease (isolated cells or small clusters); TRG 2 showed residual tumor with stromal fibrosis; and TRG 3 demonstrated limited treatment effect.

### Immunofluorescence (IF)

Cells were fixed using 4% paraformaldehyde and permeabilized with 0.2% Triton X-100. Subsequently, the fixed cells were incubated with the primary antibodies against TLR3 (#13915, Abcam; #6961, CST), PRMT5, c-Myc (#32072, Abcam; #9E10, SANTA CRUZ) or J2 (#10010200, Scicons) followed by staining with the fluorochrome-conjugated secondary antibodies: Alexa Fluor 594 anti-mouse IgG, Alexa Fluor 488 anti-rabbit IgG (Abcam). The J2 antibody acts as an anti-dsRNA antibody that specifically recognizes and binds dsRNA. Image capture was conducted using Zeiss’s LSM 5 Pascal confocal microscope (Germany) with a 60× magnification lens, operated through LSM PASCAL software (v4.2 SP1).

### Liquid chromatograph mass spectrometer

For protein quantitative mass spectrometry (Fig. S2b), nuclear and cytoplasmic fractions of Panc1 cells overexpressing TLR3 were eluted from anti-Flag M2 beads (M8823, Sigma-Aldrich) using IP lysis buffer at 98 °C for 10 min and subsequently subjected to SDS-PAGE electrophoresis, after which the gel was silver stained.

For protein quantitative mass spectrometry (Fig. 5a), Panc1 cells with mock and Flag-TLR3 overexpression were eluted from anti-Flag M2 beads (M8823, Sigma-Aldrich) using 100 μl sample buffer at 98 °C for 10 min and subsequently subjected to SDS-PAGE electrophoresis, after which the gel was silver stained.

The protein bands of interest were carefully cut from the separation gel, followed by destaining and in-gel digestion using modified trypsin (Worthington Biochemical Corporation). The resulting peptide mixtures were analyzed via nanoLC-MS/MS using a NTCC-360/75-3-105 nanospray column (Nikkyo Technos) on an EASY-nLC1200 system (Thermo Fisher Scientific). Mass spectrometry was performed on a Q Exactive HFX instrument (Thermo Fisher Scientific). The acquired LC-MS/MS data were processed using Progenesis QI software (Waters Corporation), with protein identification conducted by searching against public databases (HMDB: https://www.hmdb.ca/, LipidMaps: https://www.lipidmaps.org/) and a custom database provided by Shanghai Lu-Ming Biotech Co., Ltd.

### Flow cytometry

For apoptosis detection, single-cell suspensions of WT Panc1 cells or Panc1 cells with TLR3 KO, TLR3 rescue, NLS-TLR3 rescue, NES-TLR3 rescue were labeled with Annexin V (Annexin V Apoptosis Detection Kit I, BD Biosciences) at room temperature (RT) for 20 minutes. Subsequently, the cells were stained with propidium iodide (PI) before analysis using flow cytometry on BD LSR Fortessa (BD Biosciences). The intensity of mean fluorescence was calculated with FlowJo software on BD LSR Fortessa fluorescence-activated cell sorter (BD Biosciences).

### RNA-seq

Total RNA was isolated via the TRIzol reagent (Invitrogen, CA, USA) following the supplier’s instructions. The concentration of the isolated RNA was calculated by NanoDrop 2000 spectrophotometer (Thermo Scientific, USA), while RNA integrity was analyzed by the Agilent 2100 Bioanalyzer (Agilent Technologies, Santa Clara, CA, USA). Subsequently, the libraries were prepared using VAHTS Universal V10 RNA-seq Library Prep Kit (Premixed Version) as per the recommended protocol. Finally, transcriptome sequencing and subsequent analyses were carried out by OE Biotech Co., Ltd. (Shanghai, China).

Enrichment analyses (GO, KEGG pathways, Reactome, and Wiki Pathways) of DEGs were analyzed by the hypergeometric distribution using R (v 3.2.0). Additionally, bar charts, chord diagrams, and bubble plots of the significantly enriched terms were visualized using R (v 3.2.0).

### CUT&Tag-qPCR

CUT&Tag was performed with 3×10^5 WT and TLR3 KO Panc1 cells using Vazyme #TD904. Cells were incubated with ConA Beads Pro at RT for 10 min, and with 1 μg/ml IgG or anti-c-Myc (Abcam # ab32072) overnight. Anti-rabbit secondary antibody was used at RT for 60 min, followed by protein A/G-Tn5 at RT for 60 min. Fragmentation proceeded in TTBL buffer at 37 °C for 60 min, after which 10% SDS and 0.3 pg spike in were added and samples were incubated at 55 °C for 10 min. DNA was then extracted by DNA Extract beads. The binding of c-Myc to its downstream targets was analyzed by qPCR using primers targeting the promoter of Cst1, Snai2 and Bcl2. Enrichment was normalized to spike in and IgG controls (*n* = 3, mean ± SEM). The Primers for CUT&Tag-qPCR are listed in Table S6.

### Quantification for Western blot and IF

For Western blot, bands were quantified in ImageJ (v1.54) after background subtraction (rolling ball radius = 50 px). Protein levels were normalized to β-actin. At least three independent experimental iterations were conducted to ascertain the relative protein expression levels.

For immunofluorescence, images were captured by a Zeiss LSM 5 Pascal Laser Scanning Microscope. Background signal (from non-fluorescent areas) was subtracted. Mean fluorescence intensity (MFI) was measured in ImageJ by selecting regions of interest (ROIs) within cells and normalized to DAPI. Data were analyzed using GraphPad Prism 8.0.2 (*n* = 5 biological replicates).^72^

### Statistical analysis

Group mean comparisons were conducted with Student’s two-tailed *t* test and two-way ANOVA. The survival rate was analyzed using the Kaplan–Meier test. Immunohistochemical analysis of nuclear TLR3-positive cancer cells in primary lesions and the corresponding metastasis from pancreatic cancer patients was conducted by paired samples *t* test. All quantitative evaluations were executed using GraphPad Prism software (version 8.0.2).

## Supplementary information

Supplementary Materials

## Data Availability

Further information and requests for resources and reagents should be directed to and will be fulfilled Lead Contact, Xuetao Cao (caoxt@immunol.org). The RNA sequencing data for constructed TLR3-related Panc1 cell lines are deposited in NCBI GEO under accession code GSE299145. The mass spectrometry proteomics data for TLR3 and c-Myc are deposited in the ProteomeXchange Consortium (https://proteomecentral.proteomexchange.org) via the iProX partner repository with the dataset identifier PXD064767 and PXD064762.
