# Supplementary material for: Phosphorylated Toll-like receptor 3 nuclear translocation in cancer cell promotes metastasis and chemoresistance
Source: Signal Transduct Target Ther. 2025 Jul 18;10:225. doi: 10.1038/s41392-025-02307-7 (PMC12271568; doi:10.1038/s41392-025-02307-7)
Supplement: Supplementary file 1 — Supplementary Materials [file 41392_2025_2307_MOESM1_ESM.docx]

Supplementary Materials for

**Phosphorylated Toll-like receptor 3 nuclear translocation in cancer cell promotes metastasis and chemoresistance**

Zixin Wang^1,2^#, Yan Gu^2^#, Yanfang Liu^2,3^, Ziqiao Wang^4^, Xinyuan Chen^2^, Haoze Wang^2^, Wei Zhang^5^, Gang Jin^6^ and Xuetao Cao^1,2,4,7*^

Correspondence to: Xuetao Cao (caoxt@immunol.org)

**This PDF file includes:**

Figures S1-S9

Table S1-S6

**
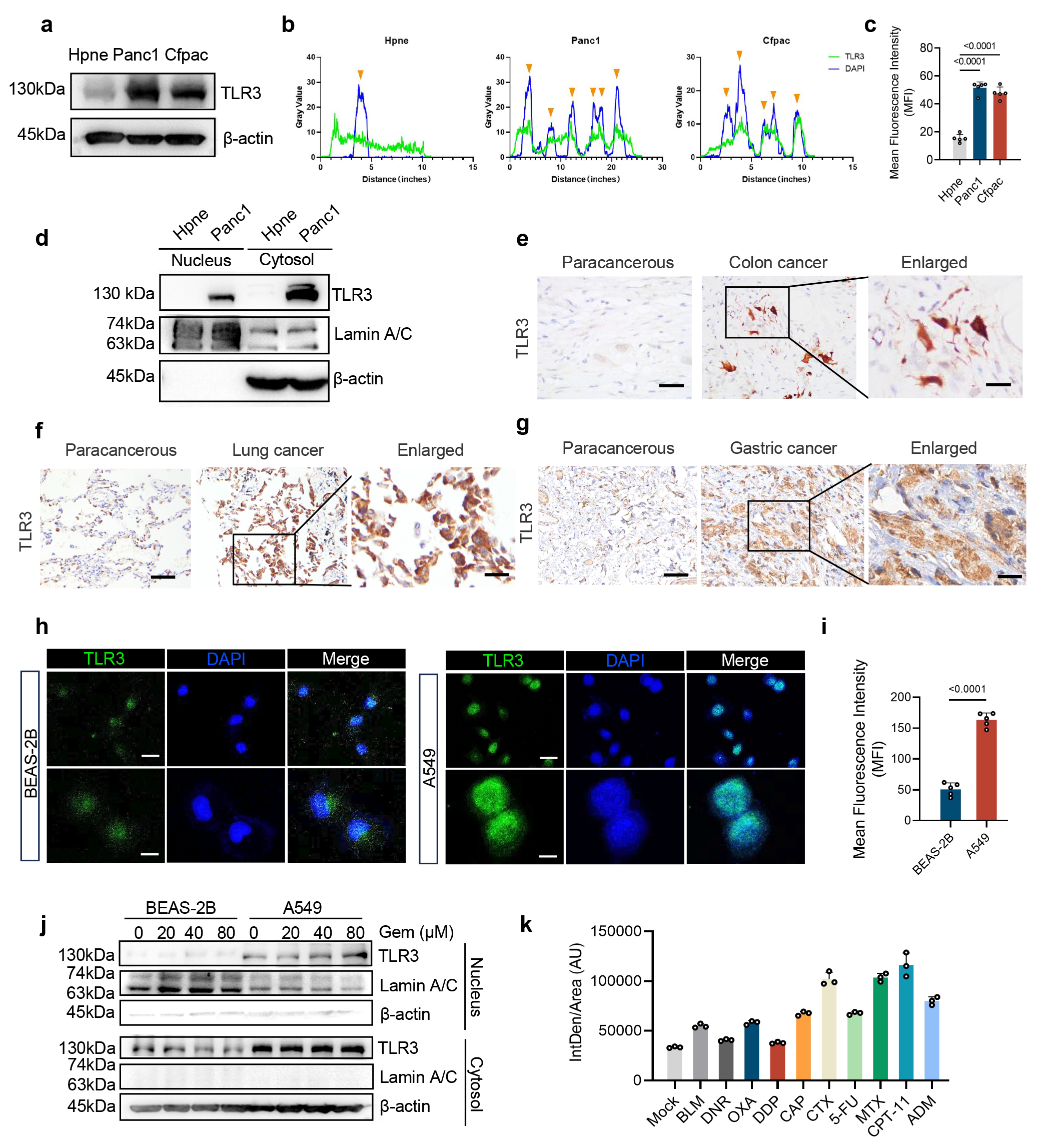
**

**Figure S1. TLR3 undergoes nuclear translocation in cancer cells upon chemotherapeutic stress.**

**a** TLR3 protein levels in Hpne, Panc1 and Cfpac cells. **b** Pixel intensity plot for nuclear TLR3 expression in Hpne, Panc1 and Cfpac cells in Fig. 1a identified by Image J. **c** Mean fluorescence intensity (MFI) of TLR3 in the nucleus of Hpne, Panc1 and Cfpac cells in Fig. 1a identified by Image J. Data shown are mean ± SD (n = 5). **d** Nuclear and cytoplasmic protein levels of TLR3, Lamin A/C and β-actin in Hpne and Panc1 cells. **e-g** Immunohistochemistry analysis of TLR3 in tumor and paraneoplastic tissues of colon cancer (**e**), lung cancer (**f**) and gastric cancer (**g**). Scale bar (left panel), 50 μm. Scale bar (right panel), 20μm. **h** Immunofluorescent analysis of TLR3 in BEAS-2B and A549 cells. Scale bar (top panel), 20 μm. Scale bar (bottom panel), 10 μm. **i** MFI of TLR3 in the nucleus of BEAS-2B and A549 cells. Data shown are mean ± SD (n = 5). **j** TLR3, Lamin A/C and β-actin protein levels in BEAS-2B and A549 cells with GEM stimulation at different concentrations (0, 20, 40, 80 μM) after nucleoplasmic separation. **k** Integrated density (IntDen) of TLR3 in the nucleus of Panc1 cells treated with different chemotherapy drugs at the recommended cellular drug dose respectively. Data shown are mean ± SD (n = 3). BLM, Bleomycin hydrochloride. DNR, Daunorubicin. OXA, Oxaliplatin. DDP, Cisplatin. CAP, Capecitabine. CTX, Cyclophosphamide. 5-FU, 5-Fluorouracil. MTX, Methotrexate. CPT-11, Irinotecan. ADM, Doxorubicin hydrochloride. Data shown are mean ± SD. Similar results were obtained from three independent experiments. One representative experiment is shown.

**
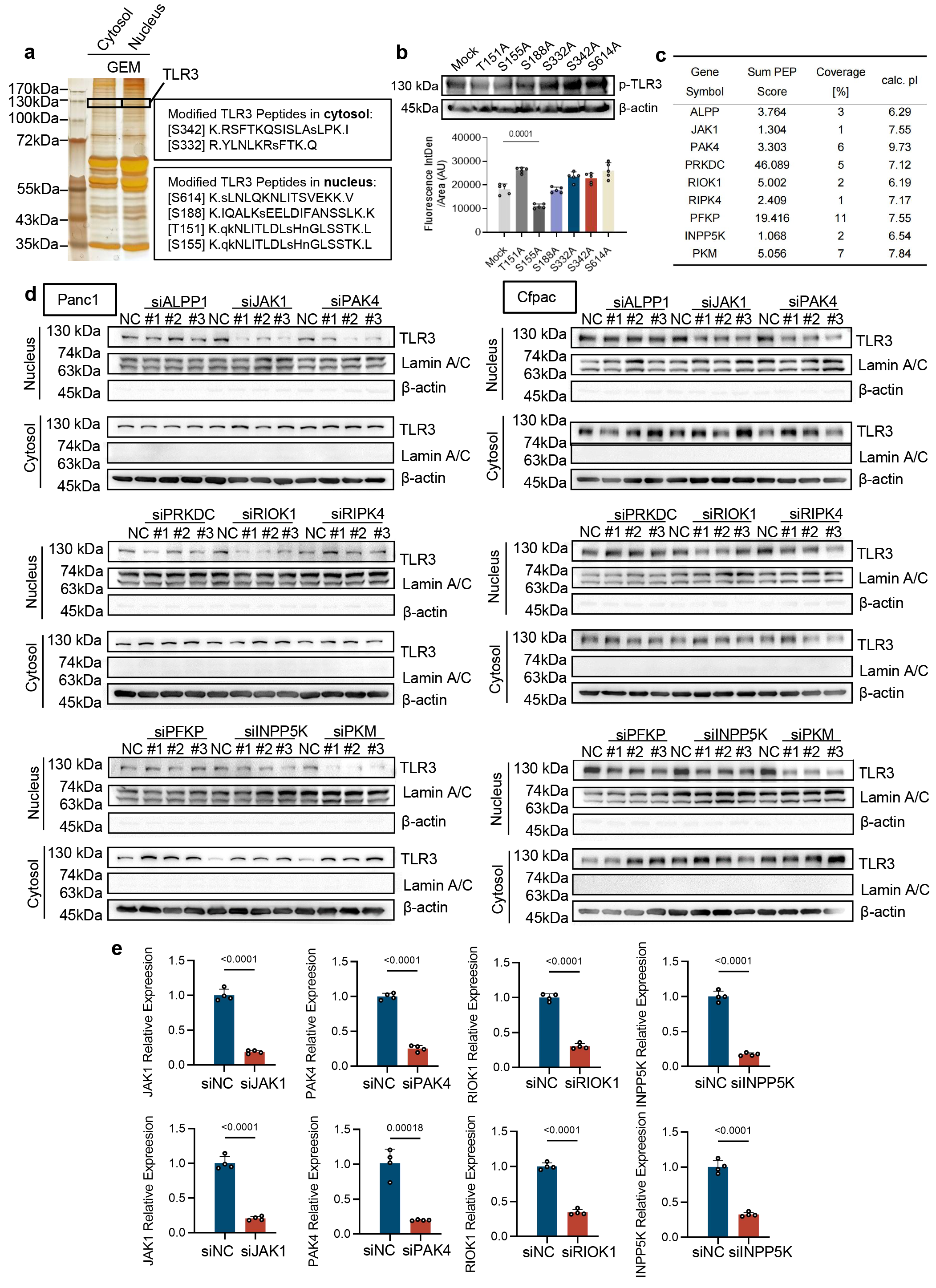
**

**Figure S2. Potential phosphokinases required for TLR3 nuclear translocation are identified in cancer cells.**

**a** TLR3 in the cytosol and nucleus of Panc1 cells treated with GEM (50 μM) for 24 h and modified TLR3 peptides in the cytosol and nucleus of Panc1 cells (detected by silver staining). **b** TLR3 phosphorylation level (top) and IntDen of nuclear TLR3 (bottom) in TLR3 knockout Panc1 cells transfected with TLR3 constructs carrying point mutations at T151, S155, S188, S332, S342 or S614 respectively. **c** Nine phosphorylation-related kinases binding to TLR3 in cytosol and nucleus of Panc1 cells treated with GEM (50 μM) for 24 h. **d** The protein level of TLR3, Lamin A/C and β-actin in cytosol and nucleus of Panc1 and Cfpac cells when silenced with ALPP1, JAK1, PAK4, PRKDC, RIOK1, RIPK4, PFKP, INPP5K and PKM respectively. **e** Analysis of JAK1, PAK4 RIOK1 and INPP5K mRNA expression respectively in WT and JAK1-silencing, PAK4-silencing RIOK1-silencing and INPP5K-silencing Panc1 cells. Data shown are mean ± SD (n = 4).

**
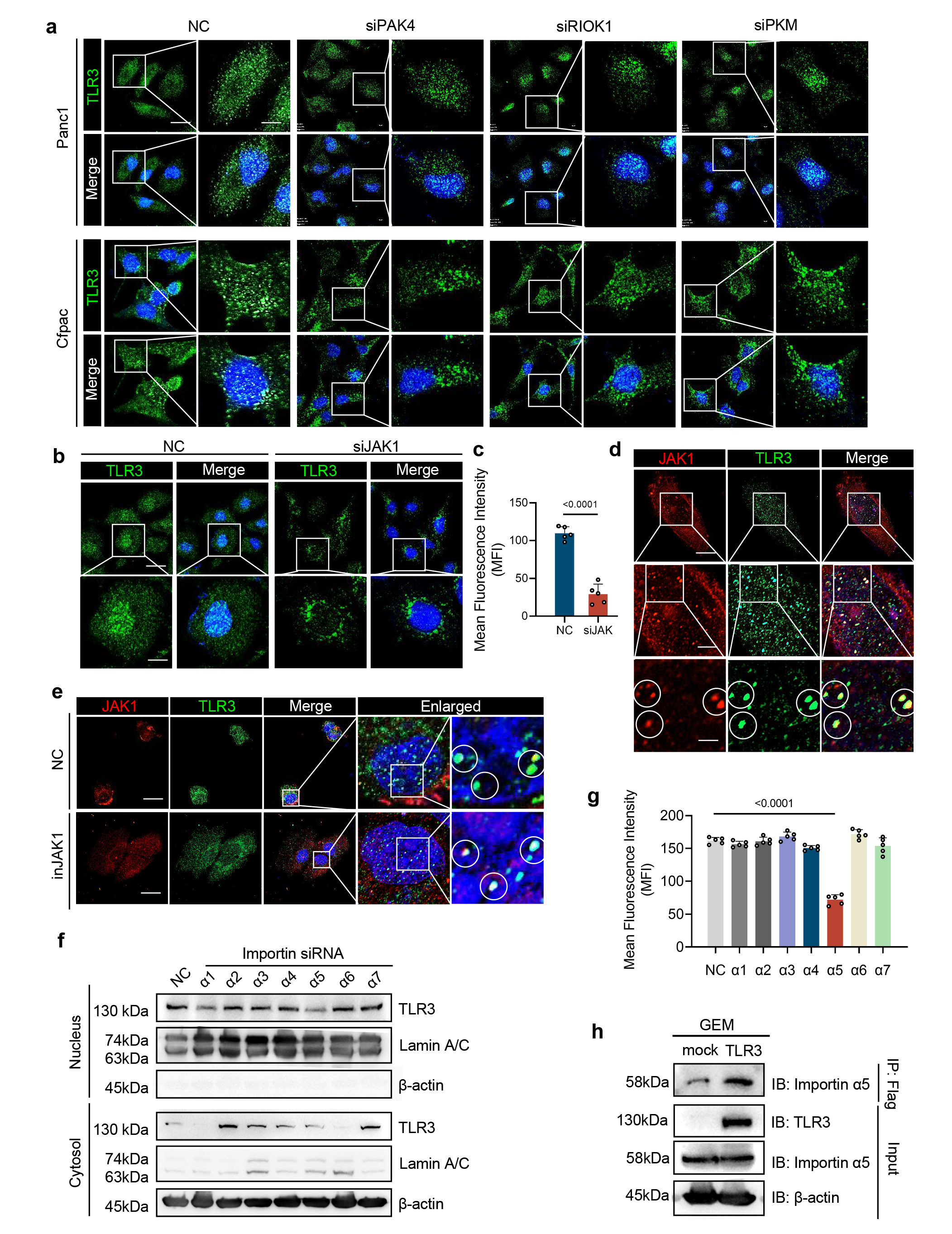
**

**Figure S3. Phosphokinase JAK1 and its kinase activity are required for TLR3 phosphorylation at S155 in cancer cells.**

**a** Immunofluorescent analysis of TLR3 expression in WT Cfpac cells, PAK4-silenced, RIOK1-silenced and PKM-silenced Cfpac cells treated with GEM (50 μM) for 24 h. Scale bar (left panel), 20 μm. Scale bar (right panel), 5 μm. **b** Immunofluorescent analysis of TLR3 in WT Cfpac cells and JAK1-silenced Cfpac cells treated with GEM (50 μM) for 24 h. Scale bar (top panel), 20 μm. Scale bar (bottom panel), 5 μm. **c** MFI of TLR3 in the nucleus of WT Panc1 cells and JAK1-silenced Panc1 cells treated with GEM (50 μM) for 24 h. Data shown are mean ± SD (n = 5). **d** Immunofluorescent analysis and co-localization of JAK1 and TLR3 in Cfpac cells. Scale bar (top panel), 10 μm. Scale bar (medium panel), 2 μm. Scale bar (bottom panel), 1 μm. **e** Immunofluorescent analysis and co-localization of JAK1 and TLR3 in Cfpac cells with or without JAK1 inhibitor treatment. Scale bar (left panel), 15 μm. Scale bar (medium panel), 3 μm. Scale bar (right panel), 1 μm. **f** The protein level of TLR3, Lamin A/C and β-actin in cytosol and nucleus of Panc1 when silenced with seven family members of importin α respectively. **g** Quantification of the mean fluorescence intensity (MFI) of TLR3 in the nucleus of Panc1 cells in Fig. 2h. Data shown are mean ± SD (n = 5). **h** Co-IP assays with anti-flag antibody and immunoblot analysis of TLR3 and Importin α5 in mock control and TLR3-flag overexpressing Panc1 cells treated with GEM (50 μM) for 24 h.


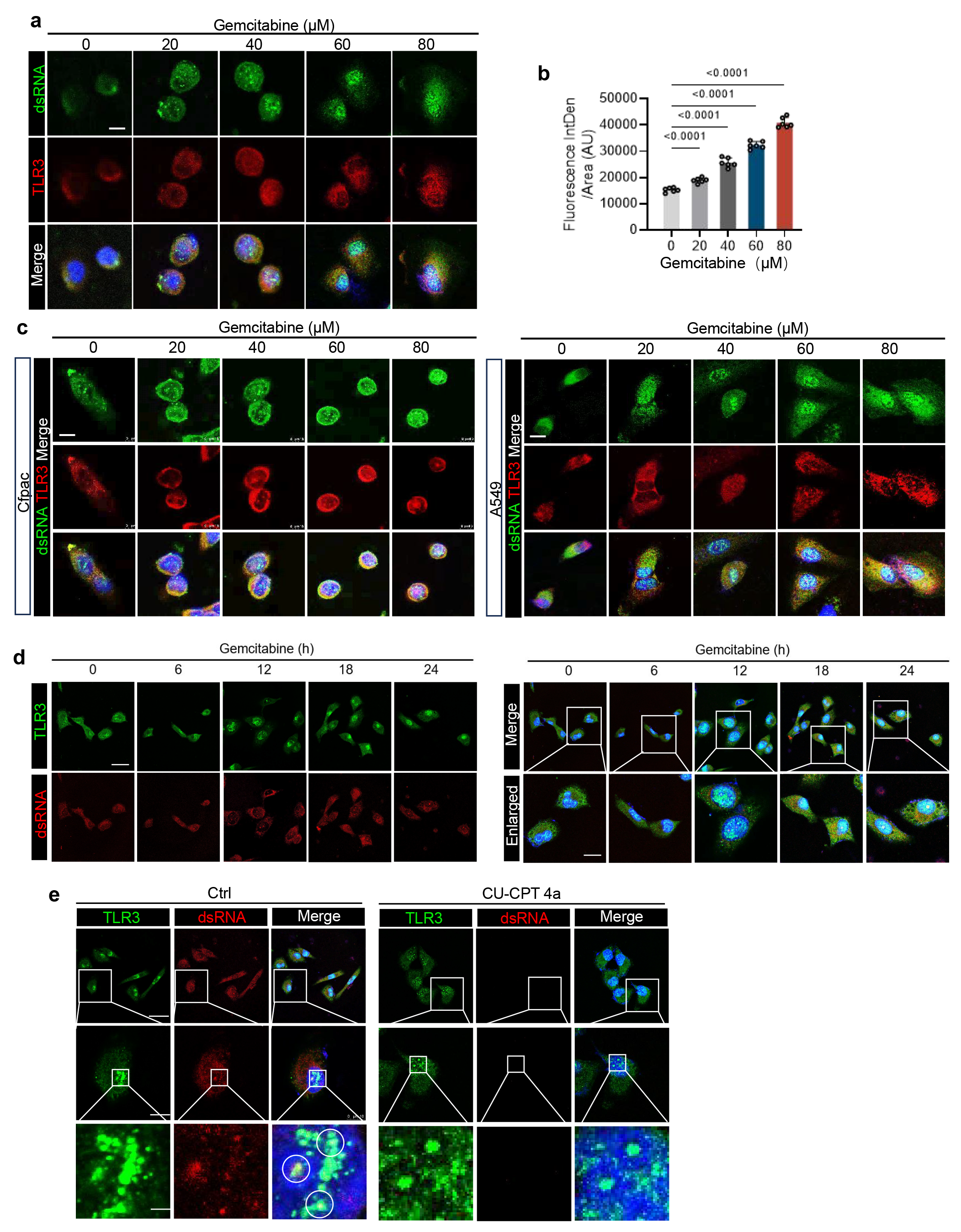


**Figure S4. dsRNA accumulation in the nucleus triggers TLR3 activation after TLR3 nuclear translocation.**

**a** The subcellular location of dsRNA and TLR3 detected by immunofluorescence microscopy in Panc1 cells with GEM treatment at 0, 20, 40, 60, 80, 100 μM for 24 h respectively. Scale bar, 5 μm. **b** IntDen of TLR3 in the nucleus of Panc1 cells treated with GEM concentrations of 0, 20, 40, 60, 80, 100 μM respectively. Data shown are mean ± SD (n = 6). Similar results were obtained from three independent experiments. One representative experiment is shown. **c** The subcellular location of dsRNA and TLR3 detected by immunofluorescence in Cfpac and A549 cells with GEM treatment at 0, 20, 40, 60, 80, 100 μM respectively. Scale bar, 5 μm. **d** Immunofluorescent analysis and co-localization of TLR3 and dsRNA in Panc1 cells with GEM treatment (50μM) at 0, 6, 12, 18 and 24h. Scale bar (top panel), 20 μm. Scale bar (bottom panel), 10 μm. **e** Immunofluorescent analysis and co-localization of TLR3 and dsRNA in Panc1 cells with or without the treatment of dsRNA inhibitor CU-CPT 4a (4μM) for 48 h. GEM (50μM) was combined in the treatment during the latter 24 h of the 48-hour period. Scale bar (top panel), 20 μm. Scale bar (medium panel), 10 μm. Scale bar (bottom panel), 0.5 μm.

**
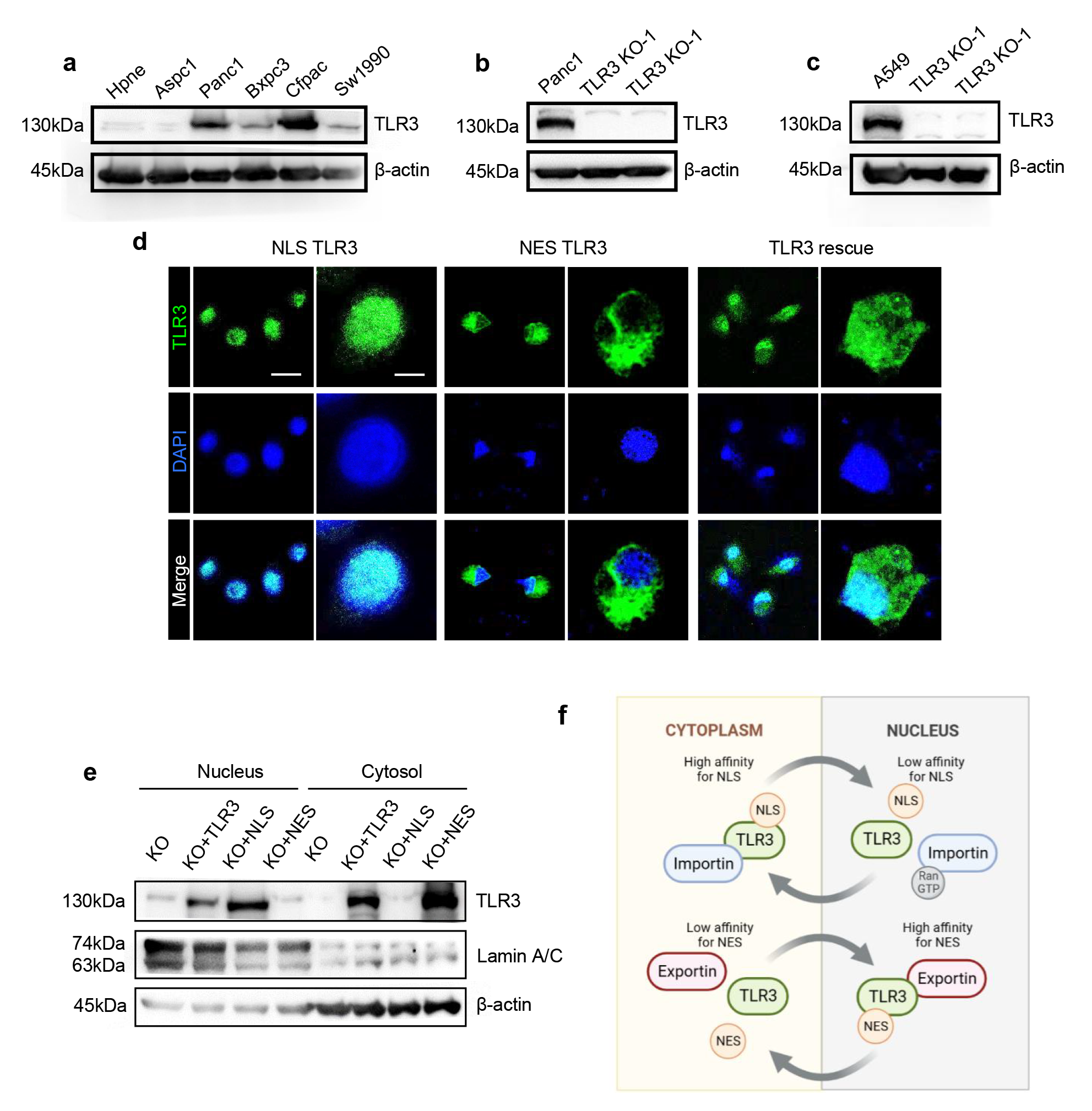
**

**Figure S5. NLS-TLR3, NES-TLR3, and wild-type TLR3 rescue are constructed in TLR3 KO cells.**

**a** Western blot analysis of TLR3 expression in Hpne, Aspc1, Panc1, Bxpc3, Cfpac and Sw1990 cells. **b, c** Western blot analysis of TLR3 in WT and TLR3 knockout Panc1 and A549 cells. **d** Immunofluorescent analysis of TLR3 in NLS-TLR3, NES-TLR3, and TLR3 rescue Panc1 cells. Scale bar (left panel), 30 μm. Scale bar (right panel), 5 μm. **e** Western blot analysis of TLR3 in NLS-TLR3, NES-TLR3 and TLR3 rescue Panc1 cells. **f** Schematic diagram showing nuclear import and export with NLS and NES sequences. Similar results were obtained from three independent experiments. One representative experiment is shown.

**
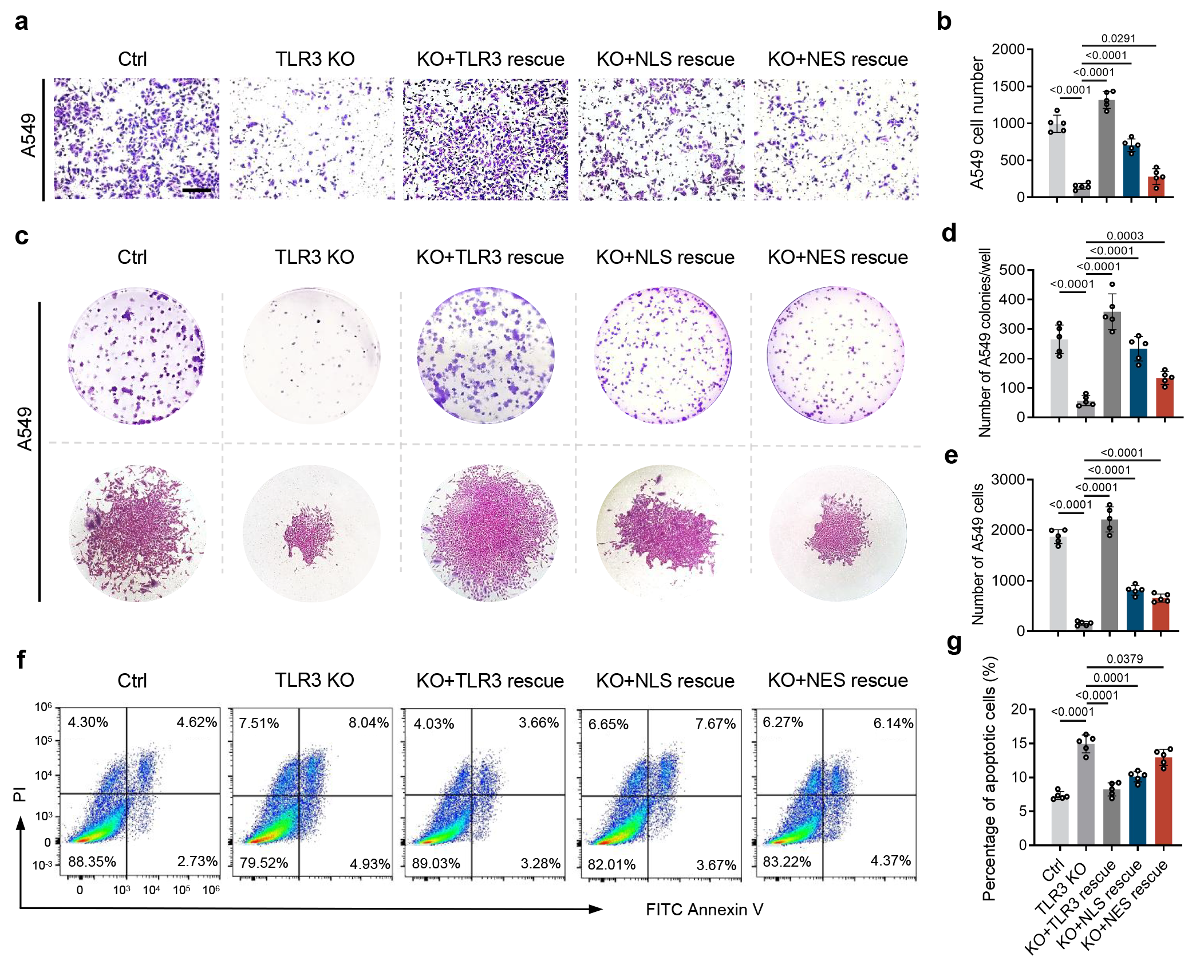
**

**Figure S6. Nuclear TLR3 promotes cancer cell proliferation and chemoresistance *in vitro.***

**a, b** Representative images (**a**) and cell numbers (**b**) for A549 cells with TLR3 knockout, TLR3 rescue, NLS-TLR3 or NES-TLR3 rescue on transwell plates. Data shown are mean ± SD (n = 5). Scale bar, 50 μm. **c, d, e** Colony-formation was determined in A549 cells with TLR3 knockout, wild-type TLR3, NLS-TLR3 or NES-TLR3 rescue. (**c**). Colony numbers are shown in the bar graph. (**d, e**) Data shown are mean ± SD (n = 5). **f, g** The proportions (**f**) and absolute number (**g**) of A549 cells with TLR3 knockout, wild-type TLR3, NLS-TLR3 or NES-TLR3 rescue after 200 μM GEM treatment were detected by flow cytometry. Numbers of apoptosis cells are shown in the bar graph. Data shown are mean ± SD (n = 5). Similar results were obtained from three independent experiments. One representative experiment is shown.

**
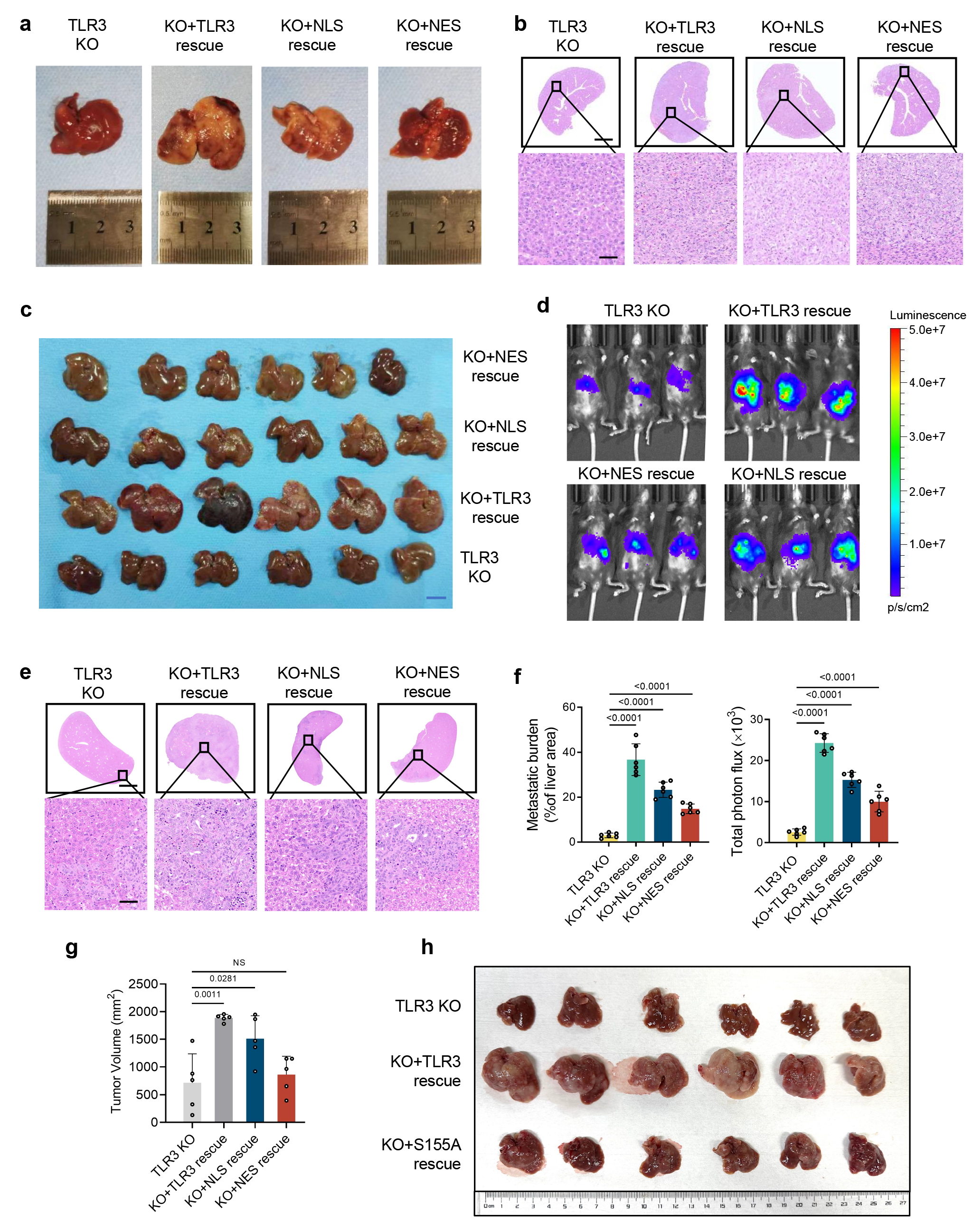
**

**Figure S7. Nuclear TLR3 promotes tumor progression and metastasis *in vivo.***

**a, b** Representative images (**a**) showing liver metastasis in NSG mice after splenic injection of Panc1 cells with TLR3 knockout, wild-type TLR3, NLS-TLR3 or NES-TLR3 rescue detected by H&E-stained liver sections (**b**). Scale bar (top panel), 2.5 mm. Scale bar (bottom panel), 50 μm. **c** Representative image of liver metastasis in NSG mice after splenic injection of Panc1 cells with TLR3 knockout, wild-type TLR3, NLS-TLR3 or NES-TLR3 rescue. Scale bar, 1 cm. **d** Representative images of liver metastasis in C57BL/6 mice with splenic injection of PancO2 cells with TLR3 knockout, wild-type TLR3, NLS-TLR3, or NES-TLR3 rescue respectively as detected by luciferase-based bioluminescence imaging. **e, f** Quantification of liver metastasis foci in C57BL/6 mice with splenic injection of PancO2 cells with TLR3 knockout, wild-type TLR3, NLS-TLR3, or NES-TLR3 rescue as detected by H&E-stained liver sections (**e**) and bar graph (**f**). Data shown are mean ± SD (n = 6). Similar results were obtained from three independent experiments. **g** Colony numbers are shown in the bar graph. Data shown are mean ± SD (n = 5). **h** Representative image of liver metastasis in NSG mice after splenic injection of TLR3 knockout, wild-type TLR3 and S155A-TLR3 rescue Panc1 cells. Similar results were obtained from three independent experiments. One representative experiment is shown.

**
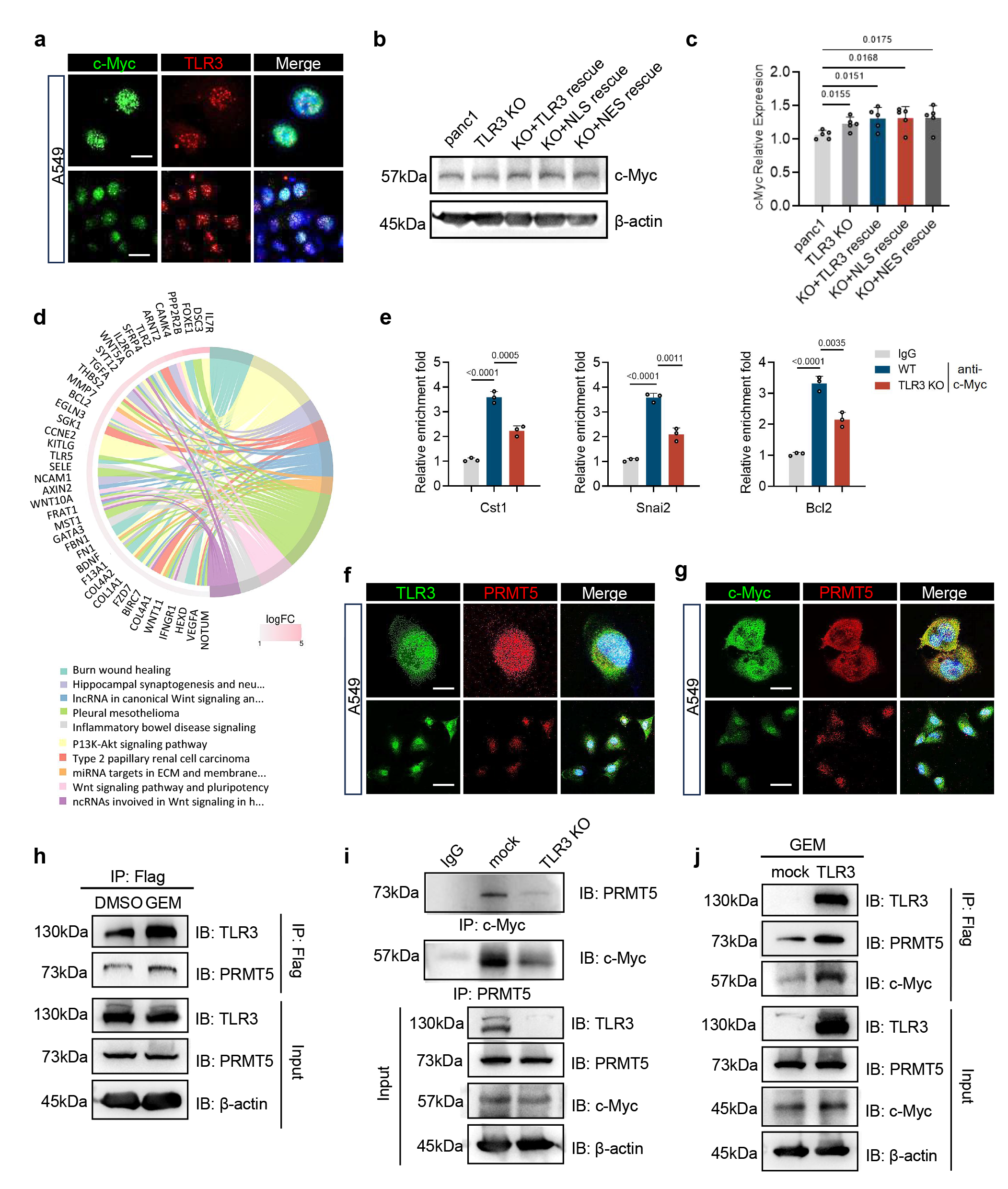
**

**Figure S8. Nuclear TLR3 mediates c-Myc dimethylation and multimerization via recruiting PRMT5 in cancer cells.**

**a** Immunofluorescent analysis of c-Myc and TLR3 co-localization in A549 cells. Scale bar (top panel), 10 μm. Scale bar (bottom panel), 20 μm. **b, c** Western blot analysis (**b**) and mRNA expression (**c**) of c-Myc in Panc1 cells with TLR3 knockout, wild-type TLR3, NLS-TLR3 or NES-TLR3 rescue. **d** Upregulated hallmarker gene sets and related signaling pathways of Panc1 cells with NLS-TLR3 rescue compared to those with NES-TLR3 rescue via WikiPathways. **e** CUT&Tag-qPCR analyses for c-Myc binding at the promoter of Cst1, Snai2 and Bcl2 in WT and TLR3 KO Panc1 cells. Data shown are mean ± SD (n = 3). **f** Immunofluorescent analysis of TLR3 and PRMT5 in A549 cells with GEM (50 μM) treatment for 24 h. Scale bar (top panel), 5 μm. Scale bar (bottom panel), 20 μm. **g** Immunofluorescent analysis of c-Myc and PRMT5 in A549 cells. Scale bar (top panel), 5 μm. Scale bar (bottom panel), 20 μm. **h** Co-IP assays with anti-flag antibody and immunoblot analysis of TLR3 and PRMT5 in TLR3- overexpressing Panc1 cells under GEM-treated (50 μM, 24 h) or untreated condition. **i** Immunoblot analysis of PRMT5 and c-Myc in WT and TLR3 knockout Panc1 cells after Co-IP with antibodies against PRMT5 or c-Myc. **j** Co-IP assays with anti-flag antibody and immunoblot analysis of TLR3, PRMT5 and c-Myc in mock control and TLR3-flag overexpressing Panc1 cells treated with GEM (50 μM) for 24 h.

**
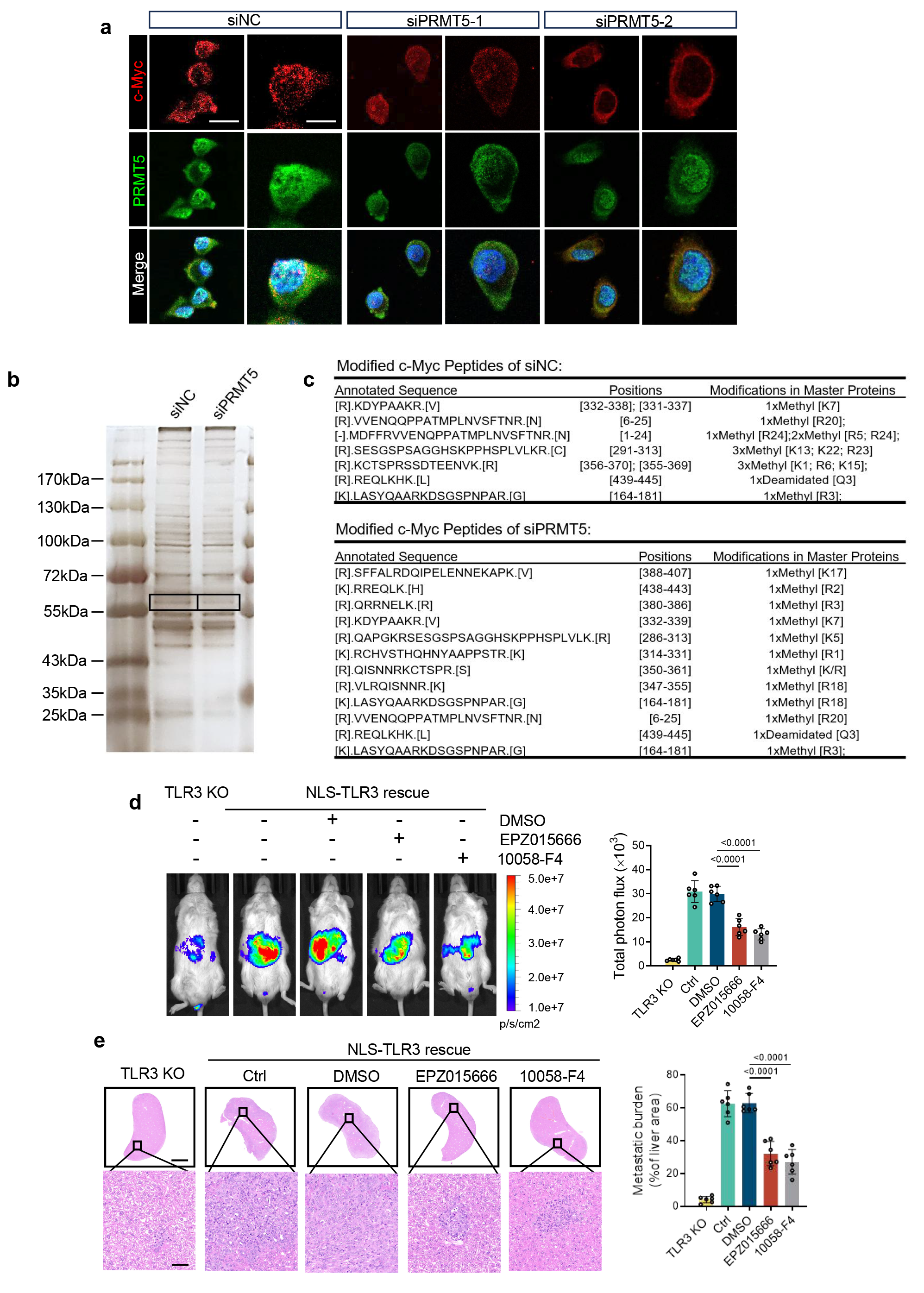
**

**Figure S9. PRMT5 facilitates c-Myc dimethylation and multimerization to promote metastasis.**

**a** Immunofluorescent analysis of c-Myc and PRMT5 in siNC and PRMT5-silenced Panc1 cells. Scale bar (left panel), 20 μm. Scale bar (right panel), 8 μm. **b** c-Myc pulled down from siNC and PRMT5-silenced Panc1 cells was visualized by silver staining. **c** The analysis of modified c-Myc peptides in siNC and PRMT5-silenced Panc1 cells after protein post-translational modification mass spectrometry. **d, e** Representative images and quantification of liver metastasis in NSG mice with splenic injection of Panc1 cells with TLR3 knockout and NLS-TLR3 rescue respectively treated with DMSO, EPZ015666 or 10058-F4 as detected by luciferase-based bioluminescence imaging (**d**), H&E-stained liver sections and bar graph (**e**). Data shown are mean ± SD (n = 6). Similar results were obtained from three independent experiments. One representative experiment is shown.

**Supplementary Tables**

**Table S1. Reagent or Resource**

| REAGENT or RESOURCE | SOURCE | IDENTIFIER |
| --- | --- | --- |
| Antibodies |  |  |
| TLR3 | Cell Signaling Technology | Cat# 6961 |
| β-actin | Cell Signaling Technology | Cat# 8457 |
| Lamin A/C | Cell Signaling Technology | Cat# 4777 |
| ChIP-Grade Protein G Magnetic Beads | Cell Signaling Technology | Cat# 9006 |
| JAK1 (6G4) Rabbit mAb | Cell Signaling Technology | Cat# 3344 |
| PRMT5 | Cell Signaling Technology | Cat# 79998 |
| Flag-tag (D6W5B) | Cell Signaling Technology | Cat# 14793 |
| c-Myc | Abcam | Cat# ab32072 |
| PRMT5 | Abcam | Cat# ab109451 |
| Goat Anti-Rabbit IgG H&L (Alexa Fluor® 488) | Abcam | Cat# ab150077 |
| Goat Anti-Mouse IgG H&L (Alexa Fluor® 594) | Abcam | Cat# ab150116 |
| Asymmetric Di-Methyl Arginine | PTM BioLab | Cat# 605RM |
| Symmetric Di-Methyl Arginine | PTM BioLab | Cat# 617RM |
| Methyl (mono) Arginine | ImmuneChem | Cat# ICP0801 |
| Dimethyl Arginine | ImmuneChem | Cat# ICP0811 |
| Dimethyl (sym) Arginine | ImmuneChem | Cat# ICP0810 |
| TLR3 | Novus Biologicals | Cat# 40C1285.6 |

| Chemicals, Peptides, Recombinant Proteins and Supplies | | |
| --- | --- | --- |
| Protein G Agarose Beads | Cell Signaling Technology | Cat# 37478 |
| Protease/Phosphatase Inhibitor Cocktail (100X) | Cell Signaling Technology | Cat# 5872 |
| FLAG® M2 magnetic beads | Sigma-Aldrich | Cat# M8823 |
| X-treme GENE**^TM^** HP | Sigma-Aldrich | Cat# 06366236001 |
| Poly (I:C) | Sigma-Aldrich | Cat# P1530 |
| Lipofectamine**^TM^** RNAi MAX Transfection | Thermo Fisher | Cat# 13778150 |
| IP Lysis Buffer | Thermo Fisher | Cat# 87788 |
| PMSF | Thermo Fisher | Cat# 8553 |
| Tris (1 M)，pH 7.0，RNase free | Thermo Fisher | Cat# AM9850G |
| EDTA (0.5 M)，pH 8.0，  RNase free | Thermo Fisher | Cat# AM9261 |
| Chemicals, Peptides, Recombinant Proteins and Supplies | | |
| NaCl (5 M)，RNase free | Thermo Fisher | Cat# AM9760G |
| DMEM/F12 | Gibco | Cat# 21331020 |
| DMEM | Gibco | Cat# 11960044 |
| Fetal Bovine Serum | Gibco | Cat# 10099141C |
| Trypsin-EDTA | Gibco | Cat# 25200-056 |
| Gemcitabine | MedChemExpress | Cat# HY-17026 |
| Bleomycin hydrochloride (BLM) | MedChemExpress | Cat# HY-17565A |
| Daunorubicin (DNR) | MedChemExpress | Cat# HY-13062A |
| Oxaliplatin (OXA) | MedChemExpress | Cat# HY-17371 |
| Cisplatin (DDP) | MedChemExpress | Cat# HY-17394 |
| Capecitabine (CAP) | MedChemExpress | Cat# HY-B0016 |
| Cyclophosphamide (CTX) | MedChemExpress | Cat# HY-17420 |
| 5-Fluorouracil (5-FU) | MedChemExpress | Cat# HY-90006 |
| Methotrexate (Amethopterin) (MTX) | MedChemExpress | Cat# HY-14519 |
| Irinotecan (CPT-11) | MedChemExpress | Cat# HY-16562 |
| Doxorubicin hydrochloride (Adriamycin) (ADM) | MedChemExpress | Cat# HY-15142 |
| Dactolisib | MedChemExpress | Cat# HY-50673 |
| Tofacitinib | MedChemExpress | Cat# HY-40354 |
| Mounting Media with DAPI - Aqueous, Fluoroshield | Abcam | Cat# 104139 |
| Denature Lysis Buffer | Sangon | Cat# C500014 |
| Nondenature Lysis Buffer | Sangon | Cat# |
| 5X Protein Loading Buffer | Sangon | Cat# C508320 |
| 5X Protein Loading Buffer (No Deturation Buffer) | Sangon | Cat# [C506032](https://www.simianti.cn/item/128345) |
| 10X Tris-Glycine SDS-PAGE Running Buffer | Sangon | Cat# 520001 |
| 10X Tris-Glycine Native PAGE Running Buffer PH 8.8 | Sangon | Cat# C506035 |
| Deoxycholic acid, sodium salt | Sangon | Cat# A600150 |
| Protein Stains K | Sangon | Cat# C500021 |
| Crystal Violet | Sangon | Cat# 600331 |
| Nonidet (R) P-40 | Sangon | Cat# 600385 |
| Dimethyl sulfoxide DMSO | Sangon | Cat# A600163 |
| Glycine | Sangon | Cat# A110167 |
| Sodium dodecyl sulfate (SDS) | Sangon | Cat# A600485 |
| Hematoxylin staining solution | Sangon | Cat# E607317 |
| Normal Goat Serum | ZSGB-Bio | Cat# ZLI-9056 |
| Antibody Dilution Buffer | ZSGB-Bio | Cat# ZLI-9030 |

| Chemicals, Peptides, Recombinant Proteins and Supplies | | |
| --- | --- | --- |
| Citrate Antigen Retrieval Solution, pH6.0 | ZSGB-Bio | Cat# ZLI-9065 |
| Tris-EDTA Antigen Retrieval Solution | ZSGB-Bio | Cat# ZLI-9079 |
| PAP Pen | ZSGB-Bio | Cat# ZLI-9303 |
| Endogenous Peroxidase Blocking Buffer | ZSGB-Bio | Cat# ZLI-9311D |
| DAB Horseradish Peroxidase Color Development Kit | ZSGB-Bio | Cat# PV-8000 |
| Phosphate Buffered Solution | ZSGB-Bio | Cat# ZLI-9063 |
| 3M Vetbond Tissue Adhesive | 3M | Cat# 1469SB |
| Critical commercial assays | | |
| NE-PER**^TM^** Nuclear and  Cytoplasmic Extraction  Reagents | Thermo Fisher | Cat# 78833 |
| PierceTM BCA Protein  Assay Kits | Thermo Fisher | Cat# 23225 |
| SuperSignal West Femto | Thermo Fisher | Cat# 34094 |
| PrimeScript™ RT Master Mix (Perfect Real Time) | TAKARA | Cat# RR036Q |
| TB Green® Premix Ex Taq ™ (Tli RNaseH Plus) | TAKARA | Cat# RR420Q |
| RNA Fast200 Cell/Tissue Total RNA Isolation Kit | Fastagen | Cat# 220011 |
| PAGE Gel Fast Preparation Kit | Epizyme | Cat# PG112 |
| PharmingenTM Annexin V-FITC | BD Biosciences | Cat# 556547 |
| ClonExpress® MultiS | Vazyme | Cat# C113-01 |
| spCas91.1/gRNA Kit | V-solid | Cat# VK001-02 |

| Experiment models: Cell lines and Mice | | |
| --- | --- | --- |
| Hpne cells | American Type Culture Collection | CRL-4023 |
| Cfpac-1 cells | American Type Culture Collection | CRL-1918 |
| Panc-1 cells | American Type Culture Collection | CRL-1469 |
| BEAS-2B cells | Beyotime | C6106 |
| A549 cells | American Type Culture Collection | CCL-185 |
| NOD.Cg-Prkdcscid IL2rgtm1Wjl/SzJ | Gempharmatech | N/A |

| Recombinant DNA | | |
| --- | --- | --- |
| pcDNA3.1(-)B-Flag | Subcloned | N/A |
| pUNO1-mcs | InvivoGen | puno-mcs |
| pCAG-T7-cas9+gRNA-pgk-Puro- T2A-GFP | V-solid | Cat# VK001-02 |

**Table S2. Primers for PCR**

| Primers for PCR | | |
| --- | --- | --- |
| m mutant T151 s | GTCAAGCAGAAGAATTTAATCG |  |
| m mutant T151 as | AAAGGGATTATTTTTAATTTTCTGGATTGAGTTGGAC |  |
| m mutant S155 s | ATCACATTAGATCTGGCTCATAATGGCTTGTCA |  |
| m mutant S155 as | TAAATTCTTCTGCTTGACAAAGGGATTATTTTTAATTTTCTG |  |
| m mutant S188 s | CAAGCGCTAAAAGCTGAAGAACTGGAT |  |
| m mutant S188 as | AATTTTATTGTTTGATAATAGAAGCTCTTGGAGATTTTCCA |  |
| m mutant S332 s | AGGTACCTGAATTTGAAACGGGCTTTTACTAAACAA |  |
| m mutant S332 as | CACATTGAAAAGCCCGTGCAAAGAG |  |
| m mutant S342 s | TCCCTTGCCGCACTCCCCAAG |  |
| m mutant S342 as | AATACTTTGTTTAGTAAAAGACCGTTTCAAATTCAGGTA |  |
| m mutant S614 s | CAGGTGTCTCTAAAGGCATTGAACCTTCAGAAG |  |
| m mutant T614 as | ATTATTAAAGACAGATGCTGGAAGTGTGTTTAAATT |  |
| h NES-TLR3-Flag s | GGATCTATTTCCGGTgaattcATGCTGCCCCCCCTGGAGCGCCTGACCCTGTCCACCACCAAGTGCACTGT |  |
| h NES-TLR3-Flag as | GGAGGGAGAGGGGCGgaattcTTACTTATCGTCGTCATCCTTGTAATCACCAGAACCACCATGTACAGAGTTTTTGGATCCAA |  |
| h NES-TLR3-Flag s | GGATCTATTTCCGGTgaattcATGCCAAAGAAGAAGCGGAAGGTCTCCACCACCAAGTGCACTGT |  |
| h NES-TLR3-Flag as | GGAGGGAGAGGGGCGgaattcTTACTTATCGTCGTCATCCTTGTAATCACCAGAACCACC ATGTACAGAGTTTTTGGATCCAA |  |
| h Flag-TLR3-NES s | GGATCTATTTCCGGTgaattcATGGATTACAAGGATGACGACGATAAGGGTGGTTCTGGT TCCACCACCAAGTGCACTGT |  |
| h Flag-TLR3-NES as | GGAGGGAGAGGGGCGgaattcTTACAGGGTCAGGCGCTCCAGGGGGGGCAGATGTACAGAGTTTTTGGATCCAA |  |

| Primers for PCR | | |
| --- | --- | --- |
| h Flag-TLR3-NLS s | GGATCTATTTCCGGTgaattcATGGATTACAAGGATGACGACGATAAGGGTGGTTCTGGTTCCACCACCAAGTGCACTGT |  |
| h Flag-TLR3-NLS as | GGAGGGAGAGGGGCGgaattcTTAGACCTTCCGCTTCTTCTTTGGATGTACAGAGTTTTTGGATCCAA |  |

**Table S3. TLR3 CRISPR guide RNA sequences**

| Primers for TLR3 CRISPR guide RNA sequences | | |
| --- | --- | --- |
| h TLR3 CRISPR guide-1 s | AAACACCG TCCAGGTACAGAATGCTACA |  |
| h TLR3 CRISPR guide-1 as | CTCTAAAAC ACTTTCGAGAGTGCCGT |  |
| h TLR3 CRISPR guide-2 s | AAACACCG CATTGCAGCTGCAACTGGCA |  |
| h TLR3 CRISPR guide-2 as | CTCTAAAAC GAATGAGCAAGTGATACAA |  |
| h TLR3 CRISPR guide-3 s | AAACACCG CAAGGAATATACCAATGCAT |  |
| h TLR3 CRISPR guide-3 as | CTCTAAAAC GAATGAGCAAGTGATACAA |  |
| h TLR3 CRISPR guide-4 s | AAACACCG AGCCATCTGCTATTAATGTT |  |
| h TLR3 CRISPR guide-4 as | CTCTAAAAC GAATGAGCAAGTGATACAA |  |

**Table S4. siRNA sequences**

| Primers for siRNA sequences | | |
| --- | --- | --- |
| h Negative Control s | UUCUCCGAACGUGUCACGUTT |  |
| h Negative Control as | ACGUGACACGUUCGGAGAATT |  |
| h ALPP-Homo-364 s | AUACAAUGUAGACAAACAUTT |  |
| h ALPP-Homo-364 as | AUGUUUGUCUACAUUGUAUTT |  |
| h ALPP-Homo-306 s | UGGGGCCUGAGAUACCCCUTT |  |
| h ALPP-Homo-306 as | AGGGGUAUCUCAGGCCCCATT |  |
| h ALPP-Homo-727 s | GAAAGUACAUGUUUCGCAUTT |  |
| h ALPP-Homo-727 as | AUGCGAAACAUGUACUUUCTT |  |
| h JAK1-Homo-3131 s | GACGGAGGAAAUGGUAUUATT |  |
| h JAK1-Homo-3131 as | UAAUACCAUUUCCUCCGUCTT |  |
| h JAK1-Homo-550 s | GCUCUGGUAUGCUCCAAAUTT |  |
| h JAK1-Homo-550 as | AUUUGGAGCAUACCAGAGCTT |  |
| h JAK1-Homo-3298 s | GGACUUGGCAGCAAGAAAUTT |  |
| h JAK1-Homo-3298 as | AUUUCUUGCUGCCAAGUCCTT |  |
| h PAK4-Homo-1593 s | CUGUCAGACUUUGGGUUCUTT |  |
| h PAK4-Homo-1593 as | AGAACCCAAAGUCUGACAGTT |  |
| h PAK4-Homo-483 s | CUGGACGAGUUUGAGAACATT |  |
| h PAK4-Homo-483 as | UGUUCUCAAACUCGUCCAGTT |  |
| h PAK4-Homo-1326 s | GUGGUAAUCAUGAGGGACUTT |  |
| h PAK4-Homo-1326 as | AGUCCCUCAUGAUUACCACTT |  |
| h PRKDC-Homo-10583 s | CCCGCAGGCUAUUGUUUAUTT |  |
| h PRKDC-Homo-10583 as | AUAAACAAUAGCCUGCGGGTT |  |
| h PRKDC-Homo-11398 s | CCUCCAGGUUAGGAUUAAUTT |  |
| h PRKDC-Homo-11398 as | AUUAAUCCUAACCUGGAGGTT |  |
| h PRKDC-Homo-11513 s | GCCGUGUGAAUAUAAAGAUTT |  |
| h PRKDC-Homo-11513 as | AUCUUUAUAUUCACACGGCTT |  |
| h RIOK1-Homo-1160 s | GCAGAUCUCAGUGAAUUUATT |  |
| h RIOK1-Homo-1160 as | UAAAUUCACUGAGAUCUGCTT |  |
| h RIOK1-Homo-373 s | GGAGGGUUAUGACGAUGAUTT |  |
| h RIOK1-Homo-373 as | AUCAUCGUCAUAACCCUCCTT |  |
| h RIOK1-Homo-1277 s | GCCAACGUCAAUGAUUUCUTT |  |
| h RIOK1-Homo-1277 as | AGAAAUCAUUGACGUUGGCTT |  |
| h RIPK4-Homo-1265 s | CCAGCAAACUGAUGAAGAUTT |  |
| h RIPK4-Homo-1265 as | AUCUUCAUCAGUUUGCUGGTT |  |
| h RIPK4-Homo-831 s | CACCUUCCAAGAAAUUACUTT |  |
| h RIPK4-Homo-831 as | AGUAAUUUCUUGGAAGGUGTT |  |
| h RIPK4-Homo-214 s | GAAGCCAAGAAGAUGGAGATT |  |
| h RIPK4-Homo-214 as | UCUCCAUCUUCUUGGCUUCTT |  |
| h PFKP-Homo-1467 s | GCUCCAUUCUUGGGACAAATT |  |
| h PFKP-Homo-1467 as | UUUGUCCCAAGAAUGGAGCTT |  |
| h PFKP-Homo-754 s | GGUGUUCCUUCCAGAAUCUTT |  |
| Primers for siRNA sequences |  |  |
| h PFKP-Homo-754 as | AGAUUCUGGAAGGAACACCTT |  |
| h PFKP-Homo-2182 s | CACCGAUGAUUCCAUUUGUTT |  |
| h PFKP-Homo-2182 as | ACAAAUGGAAUCAUCGGUGTT |  |
| h INPP5K-Homo-519 s | GAAGCUUUAUGGCUACUAUTT |  |
| h INPP5K-Homo-519 as | AUAGUAGCCAUAAAGCUUCTT |  |
| h INPP5K-Homo-307 s | GCUGCCUUUAAUGACUCGUTT |  |
| h INPP5K-Homo-307 as | ACGAGUCAUUAAAGGCAGCTT |  |
| h INPP5K-Homo-1150 s | GUCAGCUACUCUUCAACCUTT |  |
| h INPP5K-Homo-1150 as | AGGUUGAAGAGUAGCUGACTT |  |
| h PKM-Homo-1259 s | CUUGCAAUUAUUUGAGGAATT |  |
| h PKM-Homo-1259 as | UUCCUCAAAUAAUUGCAAGTT |  |
| h PKM-Homo-1366 s | CCAUAAUCGUCCUCACCAATT |  |
| h PKM-Homo-1366 as | UUGGUGAGGACGAUUAUGGTT |  |
| h PKM-Homo-1603 s | GAGAUGUGGUCAUUGUGCUTT |  |
| h PKM-Homo-1603 as | AGCACAAUGACCACAUCUCTT |  |
| h PRMT5-Homo-240 s | GGGACUGGAAUACGCUAAUTT |  |
| h PRMT5-Homo-240 as | AUUAGCGUAUUCCAGUCCCTT |  |
| h PRMT5-Homo-496 s | GGACCUGAGAGAUGAUAUATT |  |
| h PRMT5-Homo-496 as | UAUAUCAUCUCUCAGGUCCTT |  |

**Table S5. Primers for RT-qPCR**

| Primers for RT-qPCR | | |
| --- | --- | --- |
| siRNA targeting hPRMT5-1 s | CTGTCTTCCATCCGCGTTTCA |  |
| siRNA targeting hPRMT5-1 as | GCAGTAGGTCTGATCGTGTCTG |  |
| siRNA targeting hPRMT5-2 s | CGATCAGACCTACTGCTGTCA |  |
| siRNA targeting hPRMT5-2 as | CTCGGAGTTCCTGCGAATCT |  |
| siRNA targeting hPRMT5-3 s | TCAGGAAGATAACACCAACCTGG |  |
| siRNA targeting hPRMT5-3 as | AGCCACTGCAATCCTCTTACTAT |  |
| siRNA targeting hc-Myc-1 s | GGCTCCTGGCAAAAGGTCA |  |
| siRNA targeting hc-Myc-1 as | CTGCGTAGTTGTGCTGATGT |  |
| siRNA targeting hc-Myc-2 s | TCCCTCCACTCGGAAGGAC |  |
| siRNA targeting hc-Myc-2 as | CTGGTGCATTTTCGGTTGTTG |  |
| siRNA targeting hc-Myc-3 s | GTCAAGAGGCGAACACACAAC |  |
| siRNA targeting hc-Myc-3 as | TTGGACGGACAGGATGTATGC |  |
| siRNA targeting hGAPDH s | AGATCCCTCCAAAATCAAGTGG |  |
| siRNA targeting hGAPDH as | GGCAGAGATGATGACCCTTTT |  |
| siRNA targeting hJAK1-1 s | CTTTGCCCTGTATGACGAGAAC |  |
| siRNA targeting hJAK1-1 as | ACCTCATCCGGTAGTGGAGC |  |
| siRNA targeting hJAK1-2 s | CCACTACCGGATGAGGTTCTA |  |
| siRNA targeting hJAK1-2 as | GGGTCTCGAATAGGAGCCAG |  |
| siRNA targeting hPAK4-1 s | GGACATCAAGAGCGACTCGAT |  |
| siRNA targeting hPAK4-1 as | CGACCAGCGACTTCCTTCG |  |
| siRNA targeting hPAK4-2 s | ATCTGGTCGCTGGGGATAATG |  |
| siRNA targeting hPAK4-2 as | CAGGTTGTCCCGAATCATCTTC |  |
| siRNA targeting hRIOK1-1 s | GGCTAAACACAGCAGAGATACC |  |
| siRNA targeting hRIOK1-1 as | AACTCCCGAGCCTTGGATTCT |  |
| siRNA targeting hRIOK1-2 s | GGCTCGGGAGTTGTACCTG |  |
| siRNA targeting hRIOK1-2 as | CCACGGACTGAGACACGTC |  |
| siRNA targeting hINPP5K-1 s | AGGGGCGAGACATCCCAAA |  |
| siRNA targeting hINPP5K-1 as | AGTCCTCGATCCGAAAGTTCA |  |
| siRNA targeting hINPP5K-2 s | ACTTTCGGATCGAGGACTTTGG |  |
| siRNA targeting hINPP5K-2 as | GGAGCAGCGGGTCATGTTT |  |

**Table S6. Primers for CUT&Tag-qPCR**

| Primers for CUT&Tag-qRNA sequences | | |
| --- | --- | --- |
| h CST1-1 CUT&Tags | ACAGGCGACCAAGGAGTCTA |  |
| h CST1-1 CUT&Tag as | TGCCCCAGACAGTATCTTCC |  |
| h CST1-2 CUT&Tags | CTTCCACCCTCTCCTCTCCT |  |
| h CST1-2 CUT&Tag as | TCCGCAGACTGAGAGGATTT |  |
| h CST1-3 CUT&Tags | ACTTTCTGGAGCAGGCATGT |  |
| h CST1-3 CUT&Tag as | CCTGGTCAGGGTGGTCTCTA |  |
| h CST1-4 CUT&Tags | TCAGGTGAGTCTTCCAAGCA |  |
| h CST1-4 CUT&Tag as | GGGAACCCACAGGTCTCTCT |  |
| h CST1-5 CUT&Tags | TTCAATTGCTCGTGAGTTCG |  |
| h CST1-5 CUT&Tag as | AAGACGGAACCTGGATGATG |  |
| h CST1-6 CUT&Tags | GAGGGGATGGGAAGAAAGAG |  |
| h CST1-6 CUT&Tag as | GGCAGGTGTGCATTTATCCT |  |
| h SNAI2-1 CUT&Tags | AGCCATGGCGATATGTGTTT |  |
| h SNAI2-1 CUT&Tag as | GCACCTGGGTTTATGAGAGC |  |
| h SNAI2-2 CUT&Tags | AGGTGCCTACATCCGAACAA |  |
| h SNAI2-2 CUT&Tag as | CACACAAACTGGAACCTGGA |  |
| h SNAI2-3 CUT&Tags | TTATGCAACCTGACAATGCAC |  |
| h SNAI2-3 CUT&Tag as | AACAGGTGCTGGAGGAAAAA |  |
| h SNAI2-4 CUT&Tags | ACCTCACCCTCCAAACACAC |  |
| h SNAI2-4 CUT&Tag as | CAGAGAAAAGTGCATTGTCAGG |  |
| h SNAI2-5 CUT&Tags | GTGAGAGAATGTCCGGTGGT |  |
| h SNAI2-5 CUT&Tag as | CTCTAAAGGCAGGCTGATCG |  |
| h SNAI2-6 CUT&Tags | CTTCCCCCTTCCTTTTTCAA |  |
| h SNAI2-6 CUT&Tag as | GCCAGCCTCTGGTGTTAATG |  |
| h BCL2-1 CUT&Tags | CCCTGCCATTCATCATCATT |  |
| h BCL2-1 CUT&Tag as | TCCCTGAGGGCTTCATTAGA |  |
| h BCL2-2 CUT&Tags | TCCCAGCCTCTGCATTTTAT |  |
| h BCL2-2 CUT&Tag as | TGCTCCAAAGCAAGTAGCTG |  |
| h BCL2-3 CUT&Tags | ATGATTTTGCCCTGCCATT |  |
| h BCL2-3 CUT&Tag as | TCCCTGAGGGCTTCATTAGA |  |
| h BCL2-4 CUT&Tags | GGCTGACTCCAAGGTCCATA |  |
| h BCL2-4 CUT&Tag as | TACTCCTTGGGAAGGCTGTG |  |
| h BCL2-5 CUT&Tags | AAATTGCTGCACTTGGAACC |  |
| h BCL2-5 CUT&Tag as | CCCATGGGTGTTGAGAACAT |  |
| h BCL2-6 CUT&Tags | CTGGGTGACAGAGCAAAACA |  |
| h BCL2-6 CUT&Tag as | ATAAAGGCTTCCACCACAGC |  |
